# ‘Nobody could possibly misunderstand what a group is’: a study in early twentieth-century group axiomatics

**DOI:** 10.1007/s00407-017-0193-8

**Published:** 2017-07-20

**Authors:** Christopher D. Hollings

**Affiliations:** 1 Mathematical Institute, University of Oxford, Andrew Wiles Building, Radcliffe Observatory Quarter, Woodstock Road, Oxford, OX2 6GG UK; 2 The Queen’s College, Oxford, OX1 4AW UK

**Keywords:** 01A55, 01A60, 20-03

## Abstract

In the early years of the twentieth century, the so-called ‘postulate analysis’—the study of systems of axioms for mathematical objects for their own sake—was regarded by some as a vital part of the efforts to understand those objects. I consider the place of postulate analysis within early twentieth-century mathematics by focusing on the example of a group: I outline the axiomatic studies to which groups were subjected at this time and consider the changing attitudes towards such investigations.

## Introduction

In 1940, the number theorist and mathematical populariser E. T. Bell (1883–1960) wrote the following in his book *The development of mathematics*:

The first decade of the twentieth century witnessed a somewhat feverish activity in the postulational analysis of groups, in which American algebraists produced numerous sets of postulates for groups, with full discussions of complete independence. By 1910, nobody could possibly misunderstand what a group is. (Bell 1940, p. 225)

Bell was justifying, for a general reader, the study of axiom^1^ systems in mathematics (particularly in abstract algebra) for their own sake: the so-called ‘postulational (or postulate) analysis’ that, as Bell hinted, was all the rage amongst certain American mathematicians during the early years of the twentieth century. By introducing the example of a group, systems of axioms for which had seen particularly intense study, Bell sought to reinforce the view that a thorough study of the axioms that may be used to define a mathematical object is necessary for a full understanding of that object. Although as I have noted elsewhere (Hollings 2016), Bell’s stance was somewhat out of date by the time he was writing, this was nevertheless a point of view that had prevailed amongst a broad section of the American mathematical community earlier in the century. Moreover, others shared Bell’s view that the adoption of postulational methods brought a welcome rigour to algebraic considerations: see, for example, the remarks of Easton (1902, p. 44) in a bibliographical sketch of the development of the notion of an abstract group, where it is asserted that the postulational approach had resulted in ‘very exact definitions’.^2^ These positive views of such methods, however, stand in stark contrast to the modern position, in which, it is probably safe to say, postulate analysis is thoroughly unfashionable and is even oftentimes regarded with scorn as mere ‘axiomatic tinkering’. In this paper, I consider the place of postulate analysis within twentieth-century mathematics by following Bell’s lead and focusing on the example of a group: I outline the axiomatic studies to which groups were subjected in the early twentieth century and consider the changing attitudes towards such investigations.^3^

Before proceeding any further, let us first set up a ‘standard’ definition of a group, against which we may then compare the alternative definitions that are to come. Our standard definition shall be that which it is often found in introductory undergraduate courses:^4^

### Definition 1

Let *G* be a set upon which there is defined a binary operation whose result is denoted by juxtaposition of elements, viz. the result of applying the operation to $g,h\in G$ is *gh*. We say that *G* forms a *group* with respect to this binary operation if the following conditions are satisfied:

1. the binary operation is *closed*: whenever $g,h\in G, gh\in G$;
2. the binary operation is *associative*: for any $g,h,k\in G, (gh)k=g(hk)$;
3. there exists an *identity element* for the binary operation: an element $e\in G$ such that, for any $g\in G, eg=g=ge$;
4. every element of *G* has an *inverse element* with respect to the identity element named in (3): to each $g\in G$ there corresponds $g^{-1}\in G$ such that $gg^{-1}=e=g^{-1}g$.

As a point of clarity, we note that for the purposes of this paper a group will be taken to be an inherently abstract axiomatically defined object, as *specified* by Definition 1; the opposing view (as espoused, for example, by Neumann 2008, pp. 170–171) is that Definition 1 is merely a useful *description* of a structure that should more naturally be viewed in the context of collections of permutations. The reason for adopting the former point of view is that this was the way in which groups appear to have been viewed by (most of) the early-twentieth-century authors whom we will consider.

There is of course much in Definition 1 that might be criticised (indeed, for a flavour of some of the criticisms that might be levelled against this definition, see Neumann 1999, pp. 286–287). For instance, the ‘binary operation’ has hardly been defined properly; moreover, if we were to give it a proper definition, then surely we would specify that it be a mapping $G\times G\rightarrow G$, in which case condition (1) is redundant, being already built into the notion of a binary operation. Nevertheless, there are sound pedagogical reasons for including closure explicitly in the definition of a group: when we set exercises for students that ask them to verify that a given structure is a group, closure is one of the things that they must check, so it makes sense to include it with the other axioms, rather than having it as a property of the binary operation that must be verified separately.^5^ Indeed, from the pedagogical point of view, Definition 1 is probably the ‘best possible’ definition of a group (I do not attempt to quantify the notion of ‘best possible’): it gives us the basic outline of what a group is, whilst at the same time providing us with just enough information to be able easily to prove further elementary facts (uniqueness of the identity, uniqueness of inverses, the *n*-fold extension of associativity, and so on). The other versions of the group definition that we will see below usually require us to do more work before we can get to these elementary facts. This raises the question, to which I will return below, of whether this extra work is in any way edifying.

Having hinted above at the existence of ‘alternative definitions of groups’, it is perhaps beneficial for us to pause here and look at one such definition, as a taste of what is to come. We consider the ‘one-sided’ notion of a group, first developed by the American mathematician L. E. Dickson (1874–1954), who will feature heavily in Sect. 4. For the time being, we give the definition in a form similar to that used for Definition 1—the slightly more elaborate formulation used by Dickson (1905a, p. 199) will appear in Sect. 4.5.

### Definition 2

Let *G* be a set upon which there is defined a binary operation whose result is denoted by juxtaposition of elements, viz. the result of applying the operation to $g,h\in G$ is *gh*. We say that *G* forms a *group* with respect to this binary operation if the following conditions are satisfied:

1. the binary operation is *closed*: whenever $g,h\in G, gh\in G$;
2. the binary operation is *associative*: for any $g,h,k\in G, (gh)k=g(hk)$;
3. there exists a *right identity element* for the binary operation: an element $e\in G$ such that, for any $g\in G, ge=g$;
4. every element of *G* has an *right inverse element* with respect to the right identity element named in (3): to each $g\in G$ there corresponds $g'\in G$ such that $gg'=e$.

Thus, rather than postulating a two-sided identity, with respect to which there exist two-sided inverses, we pare the definition down so that we demand only a right identity and right inverses. To see that such a structure is indeed still a group, we may reproduce Dickson’s own proof:

### Proof

We must show that the postulated right identity *e* is also a left identity for any element of *G* and that any right inverse is also a left inverse with respect to *e*. We know that $g\in G$ has a right inverse $g'$ such that $gg'=e$; similarly, $g'$ has a right inverse $g''$ such that $g'g''=e$. Then

1 $$\begin{aligned} g=ge=g(g'g'')=(gg')g''=eg'', \end{aligned}$$

using which, we obtain:

$$\begin{aligned} g'g=g'(eg'')=(g'e)g''=g'g''=e. \end{aligned}$$

It follows also that $g''g'=e$. Using the latter, and also (1) again, we have:

$$\begin{aligned} eg=(ee)g=[e(g''g')]g=(eg'')(g'g)=ge=g. \end{aligned}$$

Just as Definition 2 gives a ‘right-hand’ notion of a group, we may also define a group in terms of a left identity and left inverses. The combinations left identity/right inverses and right identity/left inverses, however, do not define a group, although they do give a structure that has also seen much study, as outlined in Appendix C of the present paper. The one-sided definition of a group is one that appears only rarely in textbooks—we will return to this point in Sect. 7.

As well as deriving alternative definitions for groups, such as that given in Definition 2, the mathematicians whose work we will study here had another major issue in their sights: *independence* of axioms. The first author to address this matter directly was E. V. Huntington (1874–1952), another character who will enjoy a prominent position in Sect. 4. Indeed, the weeding out of redundancy was one of the motivations behind the derivation of Definition 2, along with a desire to provide a definition that was in some way *minimal*. The latter term is of course rather problematic. The authors of several of the early papers in the American school of postulate analysis seem to have been competing with each other to produce definitions with as few postulates as possible, and yet surely we can always reduce any set of postulates to a single one simply by taking the conjunction of all statements?^6^ However, as we will see in Sect. 4, the question of what form a postulate ought to take, and thus what ‘minimal’ ought to mean, was addressed by E. H. Moore (1862–1932) in a paper of 1902.

One is tempted to look back at the different sets of axioms that were developed for groups at the beginning of the twentieth century and to question their value: these exotic definitions rarely appear to be more useful than the ‘standard’ one given in Definition 1. The benefit of pursuing the ill-defined issue of the independence or minimality of a set of axioms is also open to doubt. However, I argue that, even at the time of publication, the authors of these alternative definitions perceived the latter’s value as lying not in the axioms themselves, but in the *process* of their discovery: the unpicking of the group definition in minute detail facilitated a deeper understanding of the abstract notion—of what groups were. Independence proofs, on the other hand, featuring examples in which the group axioms were negated in turn, aided in the understanding of what groups were *not*. Thus, although the importance of postulate analysis as a technique in abstract algebra has waned over the course of the twentieth century, the pursuit of this topic at the beginning of the century helped to cement the notion of a group, at least in the American literature. As I will argue in Sect. 7, it served as training for an abstract way of thinking, regardless of its specific results. The conclusions of this paper therefore complement those of Scanlan (1991), who contends that the work of the postulate analysts ‘served to provide both an experimental proof and a reference point for future researchers in the foundations of mathematics’ (Scanlan 1991, p. 999).^7^

I suggested above that Definition 1 provides us with ‘just the right amount’ of information about a group, at least for pedagogical purposes, and that therefore Definition 1 is to be preferred in this context to other specifications, such as Definition 2. If we were to start from Definition 2, on the other hand, then we might find it useful (or, indeed, be forced) first to prove the two-sided nature of the group before proceeding to the derivation of further properties, indicating that it might have been better simply to have started from Definition 1 in the first place. I am aware, however, of the beginnings of a contradiction here: I am suggesting that Definition 1 is ‘better’ than Definition 2 because it requires less preliminary work to be done before we can begin to explore the group concept more fully, and yet the overall theme of this paper is that such derivations from defining axioms were beneficial for the early-twentieth-century understanding of the abstract group concept. A simple response to this emerging contradiction is that the question of Definition 1 versus Definition 2 is one for the pedagogical sphere, whereas the bulk of the axiomatic work to be considered here took place in the research context. To address the contradiction in a fuller manner, however, I note that I will attempt to walk a fine line here between those modifications of the group axioms that provide insight, and those that are rather more artificial. For instance, given that abstract groups are derived from groups of permutations, and that intuitively, and speaking very loosely, there is something inherently ‘two-sided’ about the latter (perhaps linked in our perception to the natural invertibility of permutations), I would suggest that Definition 1 provides a more ‘reasonable’ way of specifying a group, since Definition 2, in its ‘one-sidedness’, takes us ‘too far away’ (at least in comparison to Definition 1) from the most natural groups of all: groups of permutations. I must acknowledge, however, that, as well as being somewhat vague, this distinction is of course a very subjective one,^8^ but one that I hope readers will gain some intuition for (and form their own opinions about) during the course of this paper. We will return to the question of ‘what to postulate?’, mostly in reference to the conditions found in Definition 1, in Sect. 3.

The structure of the paper is as follows. In Sect. 2, I outline the development of the abstract group concept in the nineteenth century, beginning in Sect. 2.1 with the first definitions of groups (of permutations) at the hands of Évariste Galois (1811–1832) and Augustin-Louis Cauchy (1789–1857). These were of course not abstract or axiomatic in any real way, but we may look to these definitions to help us to see where the first abstract ones came from: Sect. 2.2 outlines the efforts of Arthur Cayley (1821–1895) in this direction, whilst Sect. 2.3 turns to the work of Heinrich Weber (1842–1913), and others, who were the first to pin down the abstract axiomatic notion of a group at the end of the nineteenth century. In Sect. 2, the reader may detect some overlap with the article Neumann (1999), but nothing, I hope, that is unwarranted.^9^ Sect. 3 addresses the question of why the nineteenth-century authors discussed in the preceding section set down the group postulates that they did. Sect. 4 is the main part of the paper, in which we focus on the American postulate analysts and consider in some detail—greater detail than in previous treatments of this subject^10^—the sets of axioms that they derived during the heyday of postulate analysis in the first decade or so of the twentieth century. Section 5 follows with a brief treatment of a few later forays into such studies, both in the USA and elsewhere. Comments on later opinions of this topic, from both the research-oriented and pedagogical viewpoints, appear in Sect. 6, and some concluding remarks may be found in Sect. 7, where I argue for the subtle influence of postulate analysis on some aspects of (at least the presentation of) twentieth-century mathematics. Finally, three appendices provide details of further matters relating to postulate analysis that are not dealt with fully in the main body of the paper: Appendix A records the rather lengthy list of postulates that Huntington provided for a group in a paper of 1904 (this links to material found in Sect. 4.4), Appendix B outlines an alternative approach to group postulates involving a ternary relation (which also connects to Sect. 4.4), and Appendix C sketches the results of another strand of postulational investigation that may be viewed as an off-shoot of the study of groups.

## The evolution of the abstract group concept

### The first definitions

Although this paper is concerned with the axiomatic group concept, it is beneficial for us, and also in the interests of completeness, to begin our story with the earliest origins of the notion of a group in the work of Galois and Cauchy. As we know, these were not *axiomatic* definitions, but it is possible to look back upon them and extract the underlying properties that served to inspire the later abstract concept.

With the benefit of hindsight, we see that finite Abelian groups had appeared in the work of Gauss, as he studied binary quadratic forms (Katz 2009, §21.5.1), but we can hardly credit Gauss with the origination of the group concept. This accolade is usually reserved for Galois, with honourable mention given also to Cauchy. The reason that these two authors receive such credit is that the group structures that appeared in their work emerged from the study or use of collections of permutations/substitutions that were assumed to be closed under composition.^11^

In the case of the latter, it is in a paper of 1845 that we find a definition that appears to point towards the modern notion of a group. Thirty years earlier (Cauchy 1815), Cauchy had undertaken to study the permutations of the variables of a function, following up on the suspicion that these might be useful in attempts to solve higher-degree polynomial equations; the result was a theorem (together with a stronger conjecture) that placed a bound on the number of functions that may result from one of a general form upon the permutation of its variables (Stedall 2011, p. 205). In 1845, Cauchy developed the theory of permutations further. Roughly two pages into a section headed ‘Considérations générales’, we find the following definition:

Let us now consider several substitutions

$$\begin{aligned} \begin{pmatrix} B \\ A \end{pmatrix}, \begin{pmatrix} D \\ C \end{pmatrix}, \begin{pmatrix} F \\ E \end{pmatrix}, \ldots \end{aligned}$$

with respect to *n* letters $x,y,z\ldots$. I will call *derived* substitutions all those that one may deduce from the given substitutions, multiplied one or several times by one another or by themselves in any order, and the given substitutions together with the derived substitutions will form what I will call a *system of combined substitutions*.^12^

The symbols *A*, *B*, etc. denote arrangements of the *n* letters $x,y,z\ldots$, and so Cauchy’s notation is none other than the familiar ‘two-row’ form of a permutation. Indeed, Cauchy’s 1815 paper is the place where this notation originated. Note, however, that in 1845 (but not in 1815), these symbols were to be read from bottom to top: the arrangement *A* is mapped to the arrangement *B*, and so on. It is interesting to observe that Cauchy’s ‘multiplication’ of substitutions was one of the first times that such a notion had been applied to mathematical entities that were not pure numbers (see, for example, the comments in Stedall 2008, p. 354), and so we might also, in a moment of extravagance, place Cauchy on the road to the abstraction of the group concept.

Stepping swiftly back from such a bold claim, we note that there is nothing abstract or, indeed, particularly axiomatic about Cauchy’s definition of his system of combined substitutions. If we were to contrive to pull out one ‘axiom’ from the above, it could only be that of closure: the product of any two substitutions (the corresponding ‘derived substitution’) belongs to the system.^13^ Thus, on the face of it, Cauchy’s definition bears little resemblance to our Definition 1. However, as we know perfectly well, in a finite system of permutations such as Cauchy’s, if not in an abstract system, it is sufficient merely to demand closure: the other modern axioms follow.^14^ We note also at this point that Galois’ notion of a group, insofar as he provided any definition, was again simply a group of permutations, whose only explicitly stated property was that of closure under composition: see Neumann (2011, pp. 114–115).

### Cayley and abstraction

As was first shown in detail by Wussing (1969),^15^ the first attempt at an abstract definition of a group was that offered by Arthur Cayley in a paper of 1854 entitled ‘On the theory of groups as depending on the symbolic equation $\theta ^n=1$’ (Cayley 1854). Working on caustic curves,^16^ Cayley had found six distinct transformations under which such a curve is invariant, and had discovered, moreover, that this collection of transformations was (as we would say) closed under composition of functions (Stedall 2008, p. 374). Evidently recognising a connection between what he had discovered and the group notion given by Galois, Cayley attempted to pull out those properties that the two constructions had in common.

Rather than being given in a neat compact form, Cayley’s definition of a group sprawls over the first few pages of the paper, with certain of the properties that he required being noted as consequences of previously assumed conditions, when in fact they must be taken as assumptions themselves. Cayley’s definition is certainly *abstract*, but we cannot call it *axiomatic*. Whatever axioms there are in there are imposed by our latter-day knowledge, and must be teased out; there is little to suggest that there was anything axiomatic in Cayley’s thinking.

If any part of Cayley’s work is to be interpreted as an attempt at a definition, it is the following insufficient, but much-quoted, passage:

A set of symbols

$$\begin{aligned} 1, \alpha , \beta , \ldots \end{aligned}$$

all of them different, and such that the product of any two of them (no matter in what order), or the product of any one of them into itself, belongs to the set, is said to be a *group*. (Cayley 1854, p. 124)

To the word ‘group’, there is attached the following footnote:

The idea of a group as applied to permutations or substitutions is due to Galois, and the introduction of it may be considered as marking an epoch in the progress of the theory of algebraical equations. (Cayley 1854, p. 124)

Nowhere else do we find any reference to prior work, certainly not to Cauchy’s, nor do we gain any impression that Cayley truly understood Galois’ work, apart perhaps (as the footnote suggests) from recognising some degree of importance. What Cayley did with this group concept is a far cry from anything that Galois did with it.

It seems likely that Cayley drew the above group ‘definition’ directly from Galois, whose notion of group called only for the property of closure. As we have seen, this is sufficient if one is dealing, as Galois was, with a group of permutations of a finite set, but with the benefit of hindsight we know that it is not enough to define the appropriate notion of a group in an abstract setting. Further study of Cayley’s paper suggests that he may in fact have been on the brink of this realisation himself, but his phrasing is perhaps not as careful as it might have been. Associativity, for example, appears to have been taken as implicit. It is certainly noted on the first page of the paper that

$\ldots$ the symbols $\theta , \phi , \ldots$ are in general such that $\theta .\phi \chi =\theta \phi .\chi$,&c., so that $\theta \phi \chi , \theta \phi \chi \omega$,&c. have a definite signification independent of the particular mode of compounding the symbols $\ldots$ (Cayley 1854, p. 123)

but thereafter all explicit mention of associativity properties vanishes. We also encounter here one of the problems with Cayley’s definition that has been flagged by Neumann (1999, p. 290): what are these symbols $\theta , \phi$, etc.? The paper opens with them as ‘symbols of operation’, i.e., functions, so that the above statement of the associative property appears not as an assumption, but as an inherent property of the symbols under consideration. This interpretation falls away as we progress through the paper (even though the word ‘operation’ is still applied), but associativity remains, thus presenting us with a problem: are $\theta , \phi$, etc. ‘symbols of operation’, and therefore automatically associative in their composition, or are they abstract symbols, free of specific interpretation? If the former, then we may have to strip Cayley of the accolade of having first defined *abstract* groups, whereas in the latter case, we must criticise him for having failed to make the explicit assumption of associativity in the abstract setting.

Nevertheless, even if we grant Cayley associativity, we see that his definition is still not adequate to define our modern notion of a group. Again, we find an appropriate additional property in the paper, but not stated as an assumption—immediately after the above ‘definition’, Cayley wrote:

It follows that if the entire group is multiplied by any one of the symbols, either as further or nearer factor [i.e., via multiplication on the left or on the right, respectively], the effect is simply to reproduce the group $\ldots$ (Cayley 1854, p. 124)

Cayley expressed this another way by presenting a diagram that is now hailed as the original ‘Cayley table’, noting that ‘as well each line as each column of the square will contain all the symbols $1, \alpha , \beta , \ldots$’ (Cayley 1854, p. 124).^17^ This is certainly a familiar property for finite groups, but the problem is that it does not ‘follow’ from the definition that Cayley had laid down. Cayley’s ‘group’, as defined, was merely a monoid. The additional structure that emerges upon multiplication by a ‘further or nearer factor’ must be *assumed*; it cannot be *proved*—at least in the general situation that Cayley presented.^18^

I refrain from pouring too much criticism on Cayley’s paper here—further critique may be found in Neumann (1999, p. 290).^19^ We must of course be careful not to pillory Cayley for not having immediately written down a definition that in fact took several decades to coalesce. Nevertheless, his 1854 paper is rather confused in its presentation.

Cayley’s later writings on groups go little further than this initial paper of 1854; for example, whilst the 1854 paper enumerated all groups of orders 4 and 6, a later paper dealt with order 8 (Cayley 1859). The definition used barely changed, apart perhaps from becoming a little more explicit on the questions of associativity and the existence of an identity (see, for example, Cayley 1878). The symbols used by Cayley, however, remained ‘functional symbols, each operating upon one and the same number of letters and producing as its result the same number of functions of these letters’ (Cayley 1878, p. 50). On the whole, Cayley’s work on groups retained its focus on the finding of all groups of a given order.

### Late nineteenth-century postulates

Just as Cayley is invariably credited in the historical literature with having provided the initial spark for the growth of the abstract group concept, the two names that usually appear next in the story are those of Leopold Kronecker (1823–1891) and Heinrich Weber (1842–1913). Pursuing some ideas connected with the ideal complex numbers of his teacher E. E. Kummer (1810–1893), Kronecker developed the following finite structure:^20^

Let $\theta ',\theta '',\theta ''',\ldots$ be a finite number of elements such that with any two we may associate a third by means of a definite procedure. Thus, if the result of this procedure is denoted by ${\mathfrak {f}}$, then, for two arbitrary elements $\theta '$ and $\theta ''$, which may be identical, there exists a third $\theta '''$ equal to ${\mathfrak {f}}(\theta ',\theta '')$. Moreover, we require that:

$$\begin{aligned} {\mathfrak {f}}(\theta ',\theta '')= & {} {\mathfrak {f}}(\theta '',\theta ')\\ {\mathfrak {f}}(\theta ',{\mathfrak {f}}(\theta '',\theta '''))= & {} {\mathfrak {f}}({\mathfrak {f}}(\theta ',\theta ''),\theta ''') \end{aligned}$$

and if $\theta ''$ and $\theta '''$ are different, then:

$$\begin{aligned} {\mathfrak {f}}(\theta ',\theta '')\text { is different from }{\mathfrak {f}}(\theta ',\theta '''). \end{aligned}$$

We see that Kronecker’s defining conditions were precisely the commutative and associative laws, together with (the contrapositive of) the cancellation law. We know this to be a (finite) Abelian group, although Kronecker did not use (any cognate of) that word nor does he appear to have drawn any connection with the prior work of Galois, Cauchy or Cayley.

The credit for a fully formed general axiomatic definition of a group usually goes to Weber, in a paper of 1882; the first textbook definition of the same is probably due to him also (in 1896). In the paper, we find the following:^21^

*Definition.* A system *G* of *h* elements of any type, $\Uptheta _1,\Uptheta _2,\ldots ,\Uptheta _h$ is called a *group of degree h* if the following conditions are satisfied:

I. Via some rule, which is referred to as composition or multiplication, one derives from two elements of the system a new element of the same system. In symbols
  $$\begin{aligned} \Uptheta _r\Uptheta _s=\Uptheta _t. \end{aligned}$$
II. We have
  $$\begin{aligned} (\Uptheta _r\Uptheta _s)\Uptheta _t=\Uptheta _r(\Uptheta _s\Uptheta _t)=\Uptheta _r\Uptheta _s\Uptheta _t. \end{aligned}$$
III. From $\Uptheta \Uptheta _r=\Uptheta \Uptheta _s$ and from $\Uptheta _r\Uptheta =\Uptheta _s\Uptheta$ follows $\Uptheta _r=\Uptheta _s$.

Thus, Weber’s initial definition of a group corresponds to what we would call a finite cancellative semigroup, which of course we know to be a group;^22^ indeed, Weber immediately deduced the existence of an identity and of inverse elements (Weber 1882, p. 303). He explicitly cited the prior work of Kronecker in his introduction, whilst his use of the term ‘Gruppe’, although he made no comment on the matter, is suggestive of a debt to Galois and/or Cayley.

Several years later, Weber provided an outline of Galois theory in the first volume of his *Lehrbuch der Algebra* (Weber 1895, vol. I, §§146–158), during the course of which he naturally introduced the notion of a Galois group (§156), but it is at the beginning of the second volume that we find the following abstract definition:^23^

A system *P* of things (elements) of any type will become a group if the following preconditions are met:

1. A rule is given, according to which from a first and a second element of the system a completely determined third element of the same system may be derived.
$$\ldots $$
2. the associative law is assumed,
$$\ldots $$
3. It is assumed that if either $ab=ab'$ or $ab=a'b$, then necessarily, in the first case, $b=b'$, and in the second, $a=a'$.

Although the wording and notation may be slightly different, we see that, barring finiteness, this is the same as Weber’s 1882 definition—but in the immediately following passages, we see some slight modifications, beginning with the following:^24^

For finite groups, from 1., 2., 3., the consequence follows:

4. If, of three elements *a*, *b*, *c* of *P*, any two are given, then one can always determine the third in only one way such that
  $$\begin{aligned} ab=c. \end{aligned}$$

Weber next proceeded to prove this assertion before commenting, at the bottom of the same page:^25^

For infinite groups, this can no longer be inferred. For infinite groups, property 4. must be taken as an additional requirement in the definition.

Thus, the first axiomatic definition of an infinite group was due to Weber. It is easy to see that the solvability of equations that condition 4. expresses implies, and is implied by, the existence of an identity and inverses (as, indeed, Weber proved: Weber 1895, vol. II, p. 5). We will see such ‘solvability of equations’ axioms appearing in the definitions of the American postulate analysts.

Weber’s definitions of a group, however, were not the only ones to appear at the end of the nineteenth century. We might mention, for example, that given by Walther Dyck (1856–1934) in an earlier issue of the same volume of *Mathematische Annalen* in which Weber published his first definition (Dyck 1882). Dyck famously adopted a statement from one of Cayley’s papers on groups (Cayley 1878) as a motto for his own (‘A group is defined by means of the laws of combination of its symbols’: Dyck 1882, p. 1 or Cayley 1878, p. 51), and his paper does indeed begin in a similar style to that of Cayley, in that Dyck’s elements are termed ‘operations’ and are thus implicitly assumed to obey the associative law. Nevertheless, explicit mention is made of inverses and the identity in a definition that sprawls over several pages—see the comments in Neumann (1999, pp. 290–291). A further prominent author who was also inspired by Cayley was William Burnside (1852–1927):

**Definition.** Let

$$\begin{aligned} A, B, C, \ldots \end{aligned}$$

represent a set of operations, which can be performed on the same object or set of objects. Suppose this set of operations has the following characteristics.

($$\alpha $$)
The operations of the set are all distinct, so that no two of them produce the same change in every possible application.
($$\beta $$)
The result of performing successively any number of operations of the set, say $A, B, \ldots , K$, is another definite operation of the set, which depends only on the component operations and the sequence in which they are carried out, and not on the way in which they may be regarded as associated. Thus, *A* followed by *B* and *B* followed by *C* are operations of the set, say *D* and *E*; and *D* followed by *C* is the same operation as *A* followed by *E*.
($$\gamma $$)
*A* being any operation of the set, there is always another operation $A_{-1}$ belonging to the set, such that *A* followed by $A_{-1}$ produces no change in any object.

The operation $A_{-1}$ is called the inverse of *A*.

The set of operations is then said to form a *Group*. (Burnside 1897, pp. 11–12)

Thus, Burnside’s definition made explicit mention of closure, associativity and inverses; the existence of an identity is deduced shortly after the definition. As with Cayley’s definition, there are aspects of Burnside’s that we might criticise, but I will not do so here (see instead Neumann 1999, p. 292); Burnside’s definition is presented here in order to demonstrate that, in contrast to some of the earlier definitions that we have seen, inverses were beginning to appear explicitly in the group definitions of the final years of the nineteenth century.

## What to postulate?

Before moving on to consider the details of the work of the American postulate analysts, we first pause to consider a question that has not yet been asked explicitly: from where were the specific postulates that we have seen derived? In treating this question, we focus on the postulates of Sect. 2, because the problem is easily solved for the postulates of later sections: later researchers mostly obtained their postulates from some subset of the authors named in the preceding section^26^—such notions as identity and inverse elements, and the associative law, had been laid down by earlier authors, and much of the work of the twentieth-century postulate analysts revolved around simple variations of these. Our analysis here will, by necessity, be both brief and speculative, for few of the nineteenth-century authors whose work we have surveyed provided any indication of where their postulates came from.

The usual way of motivating the postulates found in our Definition 1, for example, would be to point to the fact that any group of permutations possesses these properties. However, it is worthwhile to subject this assertion to greater scrutiny. As we have observed already, the only property explicitly mentioned by Galois and Cauchy in their early notions of a group was that of closure, but even this property had the potential to be problematic as the group structure came be recognised in a range of branches of mathematics: in the context of geometrical symmetry, it might be observed, for example, that the composition of two reflections will give a rotation, and so it is necessary to form a broader notion of symmetry in order to ensure the closure of the collection of transformations under consideration. However, I am not aware of this point having caused any difficulty for nineteenth-century authors, in which case it seems reasonable to speculate that the presence of closure in the very earliest definitions of groups made it a familiar and intrinsic part of the developing theory. Indeed, well into the twentieth century, closure was still referred to by some authors as ‘the group property’; see, for example, Bôcher (1927) and Carmichael (1937).

In moving from groups of permutations to abstract groups, I suggest that the associativity property is only marginally less obvious than that of closure—it simply needed someone to extract this property from the context of functions and state it explicitly in an abstract setting. Indeed, we saw exactly this in Cayley’s early explorations of the abstract group concept (leaving aside concerns about the nature of his elements). Moreover, we also find Cayley stating explicitly that his operation is *not* commutative: ‘$\theta \phi$ is of course in general different from $\phi \theta$’ (Cayley 1854, p. 123). This observation arises easily from the context of functions. I suggest also that Cayley was in a good position to comment on both the associative and commutative laws in the abstract setting since he was well versed in the traditions of British symbolical algebra: see, for example, Crilly (2006, p. 349) and Wussing (1969, English transl., pp. 230–232). Thus, despite the shortcomings of his definition, the idea of what to postulate and what not has its origin in Cayley’s early contribution to the story. It is true that commutativity later appeared in Kronecker’s list of axioms, but this is because the context within which he was working demanded it. Subsequent group-axiomatic work, including that of the following sections, recognised that commutativity should not be a condition on groups in general, but that it is the characteristic property of a particular class of groups that deserve special attention.

We saw in Sect. 2 that explicit demands for an identity or inverse elements did not feature in the early group definitions, although they usually appeared as elementary consequences of the chosen axioms. The earliest requirement beyond closure and associativity was that of Cayley that $gG=G=Gg$ for any element *g* of a group *G*. Although he made no comment on this, we might speculate that the latter condition was Cayley’s abstract version of Galois’ comment that ‘[t]he permutation from which one starts in order to indicate substitutions is completely arbitrary’ (Neumann 2011, p. 115).^27^ Cancellation, however, seems to have been an early axiom of preference. Indeed, its origin in this context may have been Cayley’s condition: in the finite case, cancellation is a (computationally, more convenient?) consequence of the latter.^28^

With regard to the identity, it is certainly clear that the identity permutation is a useful element to have within a particular collection of permutations, but it is perhaps not obvious *a priori* that it is a *necessary* element. Nevertheless this is an easy deduction to make, and we may catch a glimpse of the growing recognition of the importance of the identity element in the name given to it by some late-nineteenth-century German authors: ‘Hauptelement’ (‘principal element’: see, for example, Weber 1882, p. 303). Apart from Cayley’s implicit assumption of its presence, the first author to include the identity element within his group axioms appears to have been James Pierpont, whom we shall meet in Sect. 4.1.

The only part of Definition 1 that it remains to consider is the presence of inverse elements. These clearly go hand-in-hand with the identity, and so as the latter became an integrated part of the group definition, this paved the way for inverses to appear there also—although recall that Burnside had stipulated the presence of inverses *without* any explicit demand for an identity (but of course this follows). The gradual introduction of inverses into group definitions accompanied the integration of the theories of finite and infinite groups (see Wussing 1969, Part III). Although specific comments are lacking, it seems reasonable to speculate that the explicit demand for inverses furnished mathematicians with a definition that could be applied equally well to both finite and infinite groups, rather than using cancellation for the former, whilst noting that it is insufficient for the latter.

Although, as already noted, the comments here on the question of ‘what to postulate?’ can only be speculative, the above sequence of changes in the view of which conditions were important for the emerging group concept seem to be reasonable. We will return to this question later in the paper, most particularly in the writings of E. T. Bell in Sect. 6.1, and in the pedagogical context in Sect. 6.2. For the time being, however, we note that this mixture of postulates was the starting point for the work of the postulate analysts. As we will see, they did not stray too far from the various axioms listed in Sect. 2 and were concerned largely with experiments^29^ over which slight variations and/or combinations of these postulates were the best ones to take; indeed, in some cases, it was not the axioms themselves that were important to the postulate analysts, but the connections between them.

## American postulate analysis

Having seen how the definitions of a group had developed at the end of the nineteenth century, and thus having described the backdrop to the investigations of the early twentieth century, we are now in a position to consider the work of the American postulate analysts. During the early years of the twentieth century, American mathematicians were still being heavily influenced by their German counterparts (indeed, many American mathematicians at this time had obtained their PhDs in Germany: see Parshall and Rowe 1994), so we would expect the algebraic researchers of the USA to be well versed in the definitions of Weber and others that were outlined in the preceding section—and indeed the references given in the papers to be cited in the present section bear this out.^30^

A German influence on the American postulate analysts is in fact visible from the start, for the main inspiration for this work came from David Hilbert’s 1899 *Grundlagen der Geometrie* and the axiomatisation of geometry contained therein.^31^ One of the main protagonists was E. H. Moore of the University of Chicago. Subsequently hailed as a great proponent of the abstract point of view (Birkhoff 1938, p. 284), and perhaps even as the first American algebraist (Bell 1938, p. 10), Moore established a seminar in Chicago whose participants set about studying Hilbert’s book (Parshall and Rowe 1994, p. 383), before going on to consider the axiom systems underpinning other familiar mathematical structures, with groups being one of the first examples to which they turned. Contributors to this study, which quickly spread beyond Chicago, included Dickson, Huntington, and O. Veblen (1880–1960).

### An American precursor: James Pierpont’s groups

As well as being able to look to the work of German mathematicians, the postulate analysts could also look back to a home-grown mathematical publication: James Pierpont’s^32^ survey of Galois theory, published in two parts at the turn of the century (Pierpont 1899–1900, 1900–1901). The first of Pierpont’s articles deals with the general theory of polynomial equations, including the notion of a Galois group (or ‘Galoisian group’, as Pierpont has it), whilst the second goes in a more abstract direction, with the introduction of the following definition (Pierpont 1900–1901, pp. 47–48):

Suppose a finite number of things or elements $T_1, T_2, \ldots , T_n$ given; we say they form a group if

- A law of composition is given whereby from $T_a$ and $T_b$, taken in a definite order, a definite $T_c$ is determined. The composition we call multiplication, and write $T_aT_b = T_c$.
- Multiplication is associative, although not necessarily commutative.
- There exists one and only one element $T_1$, for which
  $$\begin{aligned} T_1T_\kappa = T_\kappa T_1 = T_\kappa \qquad \kappa = 1, 2, \ldots , n \end{aligned}$$
  This element is called the *identical element* and is conveniently represented by 1.
- For every element $T_\kappa$ exists an element (denote it by $T_\kappa ^{-1}$) such that
  $$\begin{aligned} T_\kappa T_\kappa ^{-1} = T_\kappa ^{-1}T_\kappa = 1. \end{aligned}$$
  $T_\kappa ^{-1}$ is called the inverse of $T_\kappa$.

Thus, Pierpont provided what is essentially our Definition 1, a definition of a group that works in both the finite and infinite cases, thanks to the explicit presence of inverses. He went on to provide three examples that appear to have been designed to demonstrate a range of types of different groups: substitution groups, groups of rotations of regular bodies and multiplicative groups of nonzero integers modulo a prime. Having set up basic notions connected with groups of permutations in the first paper and the earlier parts of the second, he carried these over to the abstract case:

*Definition.* All terms used in substitution groups, as *subgroups*, *invariant subgroups*, *order*, *index*, *isomorphism*, etc., we extend to all those groups, which we call *abstract groups*. (Pierpont 1900–1901, p. 48)

In the final pages of his paper, Pierpont outlined some of the basic results of the theory of abstract groups, before returning to the study of polynomial equation with a proof of the insolubility of the general quintic in radicals. In a list of references at the end of the paper (‘for those who may wish to do collateral reading or to go deeper into the subject’: Pierpont 1900–1901, p. 55), he cited Cayley (1854), Jordan (1872–1873), Hölder (1889) and Burnside (1897) as sources for his section (§59) on abstract groups. Pierpont’s citation of the papers of Jordan and Hölder was probably in connection with the various elementary group-theoretic results that follow his definition. The former is unlikely to have influenced Pierpont’s particular definition of a group, since no definition is given there; the latter, on the other hand, contains a group definition that looks at least a little like Pierpont’s:^33^

The results developed in this part are valid for all groups that consist of a *finite* number of operations. The kind of operations is irrelevant. Only the group properties that are summarised by the following are assumed:

1. Any two operations composed (multiplied) in a definite order result in a uniquely determined operation that belongs to the same collection.
2. The associative law is valid for the composition of operations, although the commutative law need not be fulfilled.
3. From each of the symbolic equations
  $$\begin{aligned} AB=AC,\quad BA=CA \end{aligned}$$
  containing the operations *A*, *B*, *C*, it can be concluded that
  $$\begin{aligned} B=C. \end{aligned}$$

A consequence of these provisions in connection with the finiteness of the number of operations is that a so-called *identity* operation *J*, and indeed only one, is present, multiplication by which leaves all other operations unchanged, and that for every operation *A* there can be found a uniquely determined inverse operation $A^{-1}$, such that

$$\begin{aligned} AA^{-1}=A^{-1}A=J. \end{aligned}$$

Certain superficial similarities between this last definition and that given by Pierpont (the clarity over the commutative law), and between Burnside’s definition in Sect. 2.3 and that of Pierpont (use of subscripts) lead us to the speculation that Pierpont’s notion of group might be a *mélange* of those of Burnside and Hölder.^34^ The reference to Cayley does not appear to have any direct bearing on the content of Pierpont’s paper (this is the only appearance of Cayley’s name in either of Pierpont’s articles): it may simply be a token historical reference, possibly derived from Burnside.

Returning briefly to Pierpont’s bibliography, we find another item of interest listed in his section on general sources, namely Weber (1895). Indeed, Pierpont should have been intimately familiar with Weber’s textbook, having reviewed it for the American Mathematical Society (AMS) not long before the publication of the first of his two papers on Galois theory (Pierpont 1898).^35^ The thoroughness of his review, together with his evident enthusiasm for its subject,^36^ suggests that Weber may have been a further strong influence on Pierpont’s formulation of Galois theory, and on his treatment of groups in particular.^37^ Moreover, his reference in the review to the group definitions of Weber’s *papers* may be indicative of a general familiarity within the American mathematical community of the definitions in question:

The definition Weber gives for abstract groups is the familiar one employed in his various papers in the *Annalen* and the *Acta Mathematica* and, indeed, much that is in the first chapters [of the second volume] is taken from these mémoires. (Pierpont 1898, p. 213)

### First steps into postulate analysis: the definitions of E. V. Huntington

In line with the comments at the beginning of Sect. 4, the first works on postulate analysis concerned the axioms of geometry (see, for example, Moore 1902a); the first published work on postulate analysis for *algebra* appears to have been a paper of Huntington (1901–1902a),^38^ whose title ‘Simplified definition of a group’ gives us a clear and immediate indication of its purpose. Indeed, Huntington (1901–1902a), p. 296) began:

Up to the present time no attempt seems to have been made to prove the independence of the postulates employed to define a group, and as a matter of fact the definition usually given contains several redundancies [here Huntington cited Weber]. These redundancies are removed in the following note, the number of necessary postulates being reduced to three, and the independence of these three being established.

In the Introduction, we have already briefly noted the problem of assigning a sensible meaning to the phrase ‘number of postulates’. This will be addressed below in the work of Moore, so we sidestep it for the time being and consider the nature of Huntington’s investigations, where intuitive ideas as to what constitutes a postulate were at play.

As one would expect from such a work on the foundations of mathematics, Huntington took great care over his fundamental concepts, going further in this respect that any of the other authors we have seen so far. Thus, for example, at the beginning of the paper he adopted a language that had not been available, for example, to Galois or Cauchy by setting out the set-theoretic basis for what was to come (Huntington 1901–1902a, pp. 296–297):

A *class* of objects is determined when any condition is given such that every object in the universe must either satisfy or not satisfy the condition. Every object which satisfies the condition is said to *belong* to the class. $\ldots$

A class thus defined is usually called, in mathematical parlance, an *assemblage* (Menge, ensemble), every object which belongs to the class being called an element of the assemblage.

A *rule of combination* in an assemblage is any rule or agreement by which, when any two elements (whether the same or different) are given, in a definite order, some object (which may or may not itself belong to the assemblage) is uniquely determined.

$$\ldots $$

When two different symbols *x* and *y* are used to represent the same object, we indicate this fact by the notation $x=y$.

A footnote attached to the second parenthetic comment in the passage about the ‘rule of combination’ directs the reader to Huntington’s lemma 10 (see below). We note that the language used by Huntington was already quite comfortably ‘abstract’: we have ‘elements’, for instance, rather than ‘operations’. His group definition follows in a similar vein (Huntington 1901–1902a, p. 297):

Any assemblage in which the rule of combination denoted by $\circ$ satisfies the three following postulates we shall call a *group* with respect to this rule of combination:

1. Given any two elements *a* and *b*, there is an element *x* such that $a\circ x=b$.
2. Given any two elements *a* and *b*, there is an element *y* such that $y\circ a = b$.
3. If $a, b, c, a\circ b, b\circ c$, and either $(a\circ b)\circ c$ or $a\circ (b\circ c)$ are elements of the assemblage, then
  $$\begin{aligned} (a\circ b)\circ c = a\circ (b\circ c). \end{aligned}$$

Thus, Huntington defined his group using conditions (1 and 2) akin to Weber’s postulate 4 on the solvability of equations within the group. He went on to comment (Huntington 1901–1902a, p. 297):

The usual definition of a group, as given for example in Weber’s Algebra, $\ldots$ contains not only these three postulates, but also certain others, which we proceed now to deduce as consequences of our postulates 1, 2, 3, thus establishing the equivalence of the two definitions.

There then follows a sequence of ten lemmas in which Huntington proved the existence of unique left and right identities in a group, the validity of the left and right cancellation laws, and a closure property whose phrasing (and presence as a *deduction* from other assumptions) looks rather odd to anyone who has learnt that closure is an inherent property of a binary operation:^39^

10. *Whatever elements* *a* *and* *b* *may be,* $a\circ b$ *is also an element of the assemblage; that is, there is an element* *c* *such that* $a\circ b = c$.

Indeed, we might argue that in light of this last lemma, Huntington’s ‘rule of combination’ is not in fact a binary operation in the way in which we understand the term today—or at least it is not *introduced* as such, namely as a mapping $A\times A\rightarrow A$, where *A* denotes Huntington’s assemblage, although the above lemma means that this is what it turns out to be.^40^

With his lemmas established, Huntington was next able to justify the assertion that his definition is equivalent to that given by Weber: Huntington’s postulates 1 and 2, combined with the presence of unique left and right identities, are equivalent to Weber’s postulate 4, and Huntington’s postulate 3 to Weber’s postulate 2. The latter’s postulate 1 is Huntington’s tenth lemma, as quoted above, whilst Weber’s postulate 3 is simply left and right cancellation, which Huntington had proved to be valid in his system. ‘Hence’, Huntington concluded, ‘the two definitions are strictly equivalent’ (Huntington 1901–1902a, p. 298). The extremely elementary examples of groups that Huntington provided at this point (the integers under addition and the positive rationals under multiplication) remind us how new the abstract group concept still was. Further elementary examples of non-groups served to demonstrate the independence of Huntington’s three postulates, ‘by the method now commonly used in such cases’ (Huntington 1901–1902a, p. 298), namely the construction of systems in which all but one of the postulates hold.^41^ Huntington made no explicit reference here to prior instances of this ‘commonly used’ method, although he described it in another paper and cited its earlier use by both Peano and Hilbert (Huntington 1902a, p. 278).

At the end of his paper, Huntington turned his attention briefly to the question of the definition of a *finite* group, his initial conclusion being that, since any group may be defined by his three postulates, a finite group is therefore definable by four, the fourth being the assumption of finiteness.^42^ That the latter is independent of the first three is easily shown. Huntington contrasted his set of four postulates with the definition using *five* postulates ‘usually given, as for example by Weber’ (Huntington 1901–1902a, p. 300), namely that via closure, left and right cancellation, associativity, and finiteness; Huntington demonstrated that these five are also independent. The tone of the final paragraphs of Huntington’s paper suggests that he was rather pleased to have brought the number of required postulates down to four: but it had apparently not occurred to him that whilst he had taken left and right cancellation as two separate postulates, they might easily be combined into a single one, thus making the ‘usual’ definition of a finite group a four-postulate one (as indeed it was for Weber). This raises again the problem of what should constitute a postulate: Huntington’s postulates are mostly very compact affairs, consisting of simple statements, suggesting that this was a deliberate choice on his part. Left cancellation, for example, is just such a simple statement, whereas two-sided cancellation might easily be viewed as a compound statement. However, this distinction is still rather imprecise, and even if Huntington did believe that simple postulates were to be preferred, he made no explicit comment to this effect.

Huntington’s ‘simplified’ definition of a group was to spark a number of responses from other mathematicians, but not before Huntington himself had provided yet another definition in a further paper of 1902, presented to the AMS on 26 April of that year, i.e., almost exactly two months after his similar presentation of his earlier definition. Whereas the first definition seems to have been derived from Weber’s, the second was inspired by Burnside’s:

The following note contains a definition of a group expressed in four independent postulates, suggested by the definition given in W. Burnside’s Theory of Groups of Finite Order (1897). The definition presented by the writer at the February meeting contained three independent postulates, and the definition just proposed by Professor Moore [see below] contains five independent postulates. The comparison of these three definitions is therefore very striking. (Huntington 1901–1902b, pp. 388–389)

The definition then follows, without any further preamble (Huntington 1901–1902b, p. 389):

We consider here an assemblage or set of elements in which a rule of combination denoted by $\circ$ is so defined as to satisfy the following four postulates:

1. *If* *a* *and* *b* *belong to the assemblage, then* $a\circ b$ *also belongs to the assemblage.*
2. $(a\circ b)\circ c=a\circ (b\circ c)$, *whenever* $a\circ b, b\circ c, (a\circ b)\circ c$ *and* $a\circ (b\circ c)$ *belong to the assemblage.*
3. *For every two elements* *a* *and* *b* *there is an element* $a'$ *such that* $(a\circ a')\circ b=b$.
4. *For every two elements* *a* *and* *b* *there is an element* $a''$ *such that* $b\circ (a''\circ a)=b$.

We notice immediately that Huntington had abandoned his circuitous handling of the closure of his rule of combination and now simply had this as a postulate. His earlier postulates 1 and 2 had been remodelled as the new 3 and 4 with a view to capturing information not only about identity elements, but also about inverses (this presumably being what Burnside’s definition ‘suggested’). As Huntington noted, his new postulates 1, 2 and 3 give rise to the old postulate 1, whilst the new 1, 2 and 4 give the old 2; hence, the above does indeed define a group. We observe that these postulates, not too different from the earlier ones,^43^ are still of the same ‘simple’ type that Huntington seemed to be favouring. He went on (Huntington 1901–1902b, p. 389):

If we wish to distinguish between finite and infinite groups we may add a fifth postulate, either:

- *The assemblage contains* *n* *elements, where* *n* *is a positiv* [sic] *integer; or*
- *The assemblage contains an infinitude of elements.*

Huntington proceeded to demonstrate the independence of 1, 2, 3, 4 and 5b, and also of 1, 2, 3, 4 and 5a in the case where $n>2$, before concluding with the observation that his new postulates 3 and 4 might be replaced by left and right cancellation, but only in the finite case.

### A first response: E. H. Moore and his ‘three canons’

Huntington’s work set the tone for the subsequent postulate analysis of groups; his style of listing the postulates, deducing simple consequences, noting equivalence to previous definitions, and then proving independence, was one that was adopted by subsequent authors in this area. As we have seen, Moore was one such author: the last-cited paper by Huntington alluded to, but did not state, a five-postulate group definition that Moore had ‘just proposed’. Indeed, in this instance, ‘just proposed’ may mean a matter of minutes earlier. As we have noted, Huntington presented his paper ‘A second definition of a group’ to the AMS meeting of April 1902; the report of this same meeting also contains a brief account of a talk given by Moore (then president of the AMS), entitled ‘A definition of abstract groups’ (Cole 1902, p. 373). Moore subsequently wrote up this lecture for publication (the manuscript was received by the AMS on 17 September 1902), and it appeared in print, under the same title, as Moore (1902b), to which paper we now turn our attention.

As one might expect, Moore’s work followed on directly from that of Huntington; his first paragraph reads:

Dr. E. V. Huntington has recently given two different definitions of abstract groups by sets of respectively three and four independent postulates; these definitions were perhaps suggested by those given by Weber and by Burnside. (Moore 1902b, p. 485)

However, the immediately following paragraph provides a hint that Moore had in fact been interested in these definitions for some time:

Some years ago Professor J. Pierpont and I independently hit on a type of definition very desirable from the grouptheoretic [*sic*] standpoint. (Moore 1902b, p. 485)

Moore gave no reference here to his own work, so we may assume that it was never published. Quite what Moore meant by the phrase ‘very desirable from the grouptheoretic standpoint’ is open to debate, but I would suggest that this is a reference to the fact that Pierpont’s definition could be applied both to finite and to infinite groups. The appearance of the compound word ‘grouptheoretic’ also opens up some speculation as to the origins of Moore’s investigations: does it point to a German influence (cf. the German ‘gruppentheoretisch’), or is it simply a typo? Its appearance later in the paper, more than once, would seem to be evidence against the latter.

I have already indicated that the problem of what should constitute a postulate is one that Moore attempted to tackle, and his efforts in this direction began with the paper under consideration: in the first of the passages quoted above, a footnote is attached to the word ‘three’, which reads:

In fact, four, for the third postulate consists of two parts, each of which is used in the development of the theory. (Moore 1902b, p. 485)

Recall that in Huntington’s three-postulate definition of a group, the third is a form of the associative law, in which ‘either $(a\circ b)\circ c$ or $a\circ (b\circ c)$’ is an element of the set in question (Huntington not having postulated closure); it appears that Moore advocated breaking this postulate into two: one part each for when $(a\circ b)\circ c$ or $a\circ (b\circ c)$ belongs to the set.^44^ Moore elaborated on the issue of the form of postulates later in the paper, but, in most other respects, he followed the pattern already laid down by Huntington: formulation of definition, proof of independence, relation to other definitions. Moore’s first definition was one by five postulates, but in the final parts of the paper he gave a further definition in terms of *six*:

$\ldots$ I am led to a slightly modified definition $\ldots$ by means of six postulates, which, even from the standpoint of abstract logic, seems to me simpler than either of those of Dr. Huntington. (Moore 1902b, p. 485)

Moore’s comments on this latter definition will bring us back to the discussions of the Introduction concerning a ‘best possible’ group definition.

Moore introduced his five-postulate definition as follows (Moore 1902b, pp. 485–486):

We have for consideration a set of elements and a multiplication-table or rule of combination whereby to every two elements *a*, *b* taken in the definite order *a*, *b* there corresponds a definite so-called product, in notation $a\circ b$, or, when without confusion, more simply, *ab*; this product may or may not be an element of the set. This set of elements, as related by the multiplication-table, constitutes a group in case the following postulates are fulfilled, viz.,

(1): For every two elements *a*, *b* the product *ab* is an element of the set.
(2): The associative law is fulfilled, that is, $( ab ) c = a ( bc)$, for every three elements *a*, *b*, *c* such that the products *ab*, *bc*, (*ab*)*c* and *a*(*bc*) are elements of the set.
($$3_l$$)
There exists a left-hand identity element, that is, an element $i_l$ such that, for every element $a, i_la=a$.
($$3_r$$)
There exists a right-hand identity element, that is, an element $i_r$ such that, for every element $a, ai_r = a$.
($$4_l$$)
If there exists a right-hand identity element, then for some such element $i_r$ it is true that for every element *a* there exists a left-hand reciprocal element, that is, an element $a_l'$ such that $a_l'a=i_r$.

To these five postulates of definition of abstract groups in general the addition of a sixth postulate (5$_\alpha$) or (5$_\beta$) serves to discriminate between the groups of the various finite orders and those of infinite order: $N= n, N = \infty$; viz.:

($$5_\alpha $$)
The number of elements is a certain finite integer *n*.

or

($$5_\beta $$)
The set contains an infinitude of elements.

Note that the fact that Moore’s *ab* ‘may or may not be an element of the set’ means that his operation is not *a priori* a binary operation in our sense, although his postulate (1) takes care of this. Notice also the slightly peculiar phrasing of his postulate (2): the preceding postulate ensures that *ab*, *bc*, (*ab*)*c* and *a*(*bc*) are all elements of the set, so the ‘such that’ seems misplaced.^45^ To compare the above with our Definition 1, we see that the only thing missing from Moore’s definition is the demand for left inverses (with respect to a right identity, for symmetry with ($4_l$))—but, as we know, this follows from the conditions given. It is also easy to show that in fact $i_l$ and $i_r$ are uniquely determined and that $i_l=i_r$; these results are amongst the ‘auxiliary theorems’ that Moore proved in his following section. Other such theorems include (Moore 1902b, p. 487):

(9$$'$$, 9$$''$$)
For every two elements *a*, *b* there exists one (theorem $9'$) and only one (theorem $9''$) element *x* such that $ax=b$.
(10$$'$$, 10$$''$$)
For every two elements *a*, *b* there exists one and only one element *y* such that $ya=b$.
(11$$'$$, 11$$''$$)
For every two element *a*, *b* there exists an element *z* and there exists an element *w* such that $(az)b=b, b(wa)=b$.

Note that, separated from (9$'$), (9$''$) corresponds to left cancellation; similarly, (10$''$) is right cancellation.

With the above postulates appropriately labelled, Moore was able to embark upon the most systematic comparison of group definitions that we have seen so far; to begin with, he gave labels to the following systems of postulates (Moore 1902b, p. 487):

$$\begin{aligned} \begin{array}{lll} (W_1) &{} : &{} (1, 2, 9', 9'', 10', 10''); \\ (W_2)_{N=n} &{} : &{} (1, 2, 9'', 10'', 5_\alpha ); \\ (H_1) &{} : &{} (2', 9', 10'); \\ (H_2) &{} : &{} (1, 2, 11', 11''), \end{array} \end{aligned}$$

where (2$'$) is essentially the same as Huntington’s original associativity postulate (Moore 1902b, p. 485):

(2$$'$$)
For every three elements *a*, *b*, *c* such that the products *ab*, *bc* and either (*ab*)*c* or *a*(*bc*) are elements of the set the associative law is fulfilled.

It is not too difficult to see that ($W_1$) is Weber’s 1896 textbook definition of a group, whilst ($W_2$) is its slight modification in the finite case; ($H_1$) and ($H_2$) are the two definitions given by Huntington. As Moore remarked, Huntington had previously shown the equivalence (in Moore’s notation) of ($W_1H_1; H_2$) in general, and of $(W_2; H_1; H_2)_{N=n}$ in the finite case; he had also shown the independence of the postulates in all instances.

To the above list of group definitions, Moore next added the following:

$$\begin{aligned} \begin{array}{lll} (W_1') &{} : &{} (1, 2, 9', 10'); \\ (M) &{} : &{} (1, 2, 3_l, 3_r, 4_l); \\ (M') &{} : &{} (1, 2, 3_l, 3_r, 4_l, 4_r); \\ (P) &{} : &{} (1, 2, 3, 3^*, 4, 5_\alpha ), \end{array} \end{aligned}$$

where (3) postulates the existence of a two-sided identity, (3$^*$) its uniqueness and (4) asserts the existence of two-sided inverses with respect to that identity; these last three labels appear only in a footnote by way of enabling Moore to write down Pierpont’s (and his own earlier) definition in the same form as the others, namely (*P*). Definition (*M*) is of course the definition with which Moore began his 1902 paper: definition ($M'$) with the redundant postulate (4$_r$) removed.

Moore provided some commentary on these various definitions; for example, he claimed that

$\ldots$ ($W_1'$) is from the grouptheoretic [*sic*] standpoint a more convenient modification of $\ldots$ ($W_1$) than is ($H_1$). (Moore 1902b, p. 487)

In order to assess this assertion, we must first unpack the definitions: Weber’s original ($W_1$) is via closure, associativity, and left and right cancellation, whilst Huntington’s reformulation ($H_1$) involves a slightly modified associativity, and solvability in the group of equations $ax=b$ and $ya=b$; Moore’s alternative reformulation ($W_1'$) calls upon closure, associativity and solvability of equations and thus eliminates the peculiarity that surrounds closure in ($H_1$). Indeed, to return to the discussion in the Introduction, Huntington’s definition ($H_1$) can perhaps be viewed as an example of an unhelpful modification of the group definition: we must do extra (unedifying?) work to prove closure before we can proceed to using the group concept in a deeper manner; to pick up on another point from the Introduction, the dropping of closure as an explicit axiom (without having built it into the definition of the binary operation instead) might be said to be taking us ‘too far away’ from groups of permutations.^46^ A question of aesthetics also lies behind this issue: do we prefer a pared-down definition such as ($H_1$), or do we baulk at the slightly fiddly postulate (2$'$)? The restoration of closure as an explicit postulate in ($W_1'$) suggests that a reasonable interpretation of Moore’s ‘$\ldots$ from a grouptheoretic standpoint $\ldots$’ in the above quotation would be that he took the latter view. Indeed, Moore’s choice in this regard may have been informed by pedagogical considerations similar to those noted in the Introduction; there is an indication within the paper that Moore had been experimenting with different definitions in his teaching: he tells us (Moore 1902b, p. 488) that in his lectures of Autumn 1900, he had employed the definition ($M'$), whereas a little earlier, in January 1897, he had defined a group by the system of postulates (1, 2, 3, 4), that is to say, our Definition 1.

The switch from ($M'$) to (1, 2, 3, 4) might be explained by Moore’s general approach to the ‘proper’ form of postulates. We have already noted his view that Huntington’s associativity postulate is a compound statement; he made the same observation about his own (2$'$): ‘[t]his is a double statement’ (Moore 1902b, p. 485, footnote §). Following this same principle, the postulates (3) and (4) might therefore be viewed as ‘double statements’ which it is preferable to break down into simpler statements like (3$_l$), (3$_r$), (4$_l$) and (4$_r$). Following his discussion of the interrelations between the above definitions, Moore acknowledged the inherent difficulties involved in making such judgements:

From the standpoint of abstract logic the canons of relative simplicity of equivalent definitions by sets of postulates are not well established. Perhaps the only established canon is this, that a definition is simplified by the omission of a group of postulates logically deducible from the remaining postulates. One is tempted to add this, that every postulate of a desirably simple definition shall be a simple statement, that is, a single and not a multiple statement. The difficulty here would arise in the precise formulation of the terms of this second canon, especially in view of the fact that the same statement may be made in various forms. At least, a definition is simplified by the substitution, for a postulate consisting of an aggregate of independent statements, of those statements as distinct postulates. (Moore 1902b, pp. 488–489)

Thus, Moore seems to have justified his own choices of postulates.^47^ He went on to cast doubt on the benefits of adopting a condensed definition such as ($H_1$), using language that again fits well with the comments made in the Introduction:

Further one may add as a third canon this, that of two definitions one with the smaller number of postulates is the simpler. As to this canon the case now in question seems to show that the definition with the larger number of independent postulates may reveal more immediately the fundamental properties of the object of definition. It is, of course, evident that the task of proving the independence of the postulates presumably increases with the number of postulates. (Moore 1902b, p. 489)

Moore proceeded to apply his ‘three canons’ to the definitions ($W_1'$), ($H_1$), ($H_2$) and (*M*). With regard to the first canon, the last three definitions are independent, this having been shown for ($H_1$) and ($H_2$) by Huntington, and for (*M*) by Moore in the final section of his 1902 paper; Moore noted, however, that the independence of ($W_1'$) remained open. In connection with simplicity of postulates, Moore reasserted that (2$'$) breaks down into two parts, which he denoted (2$_1'$) and (2$_2'$), giving rise to the new system of postulates

$$\begin{aligned} \begin{array}{lll} (H_1')&:&(2_1', 2_2', 9', 10'), \end{array} \end{aligned}$$

whose independence is easily established. This canon of simplicity, however, rather complicates matters for the other definitions, for Moore insisted that postulate (1) must break down into an ‘aggregate of statements’ (Moore 1902b, p. 489)

(1)$$_{a,b}$$
The product *ab* of the two elements *a*, *b* is an element of the set.

With the exception of (3$_l$), (3$_r$) and (4$_l$), all other postulates in ($W_1'$), ($H_1'$) must break down in a similar manner, because, to employ language not used by Moore, they are simple universally quantified statements. The intricacies of balancing Moore’s three canons are illustrated by his observation that if we break down (1) into (1)$_{a,b}$, and so on, then we lose independence: for example, for any *c*, postulate (9$'$)$_{ca,cb}$ depends on (1), (2) and (9$'$)$_{a,b}$. For a nontrivial group, we also go in dramatic fashion against the third canon: that a smaller number of postulates is to be preferred—but, as the above quotation suggests, this is not a canon that Moore was fully committed to upholding.

The three postulates (3$_l$), (3$_r$) and (4$_l$) that do not break down in the indicated manner were deemed by Moore to be of ‘greater intrinsic complexity’ (Moore 1902b, p. 489), and so he sought to replace them by postulates that he considered simpler (Moore 1902b, pp. 489–490):^48^

(3$$''$$)
There exists at least one idempotent element, that is, an element *i* identical with its square, $ii =i$.
(3$$_l''$$)
Every idempotent element is a left-hand identity element; that is, for every element *a* and every idempotent element $i, ia = a$.
(3$$_r''$$)
Every idempotent element is a right-hand identity element.
(4$$_r''$$)
For every element *a* with respect to every idempotent element *i* there exists a left-hand reciprocal element, that is, an element $a_l^{(i)}$ such that $a_l^{(i)}a=i$.

Together, (3$''$), (3$_l''$) and (3$_r''$) are equivalent to (3$_l$) and (3$_r$), whilst (4$_l$) may be replaced by (4$_l''$) within this framework. Moore was thus able to write down yet another new definition of a group:

$$\begin{aligned} \begin{array}{lll} (M'')&:&(1, 2, 3'', 3_l'', 3_r'', 4_l''), \end{array} \end{aligned}$$

which is the six-postulate definition advertised at the beginning of his paper. Indeed, Moore’s preference for his new definition ($M''$) is clear: in spite of the loss of independence through the decomposition of universally quantified postulates in the above manner, the postulates employed in ($M''$) are of the simple kind that Moore preferred. He asserted, moreover, that (3$_l''$), (3$_r''$) and (4$_l''$) may now also be decomposed in the desired manner, although it is difficult to see how, and he did not elaborate. The final justification of definition ($M''$) came, however, when Moore applied a modified version of his third canon to the various definitions under consideration: rather than seeking to minimise the number of postulates involved in a definition, he sought instead to minimise the work required to verify each set of postulates:

Finally, it seems worth while in a fairly definite way to apply the third canon of comparison to the four definitions. As a unit-operation of the determination that a given set of *N* elements with a given multiplication-table constitutes a group we consider the reading from the table of a single entry *ab*, with necessary checking. (Moore 1902b, p. 491)

Thus, for example, to check the closure of a given system of *N* elements requires $N^2$ operations; the verification of associativity requires $4N^3$ operations.^49^ The number of operations required to check some of the other postulates falls into a range, rather than being a definite figure: (3$''$) might require only one operation, if the element that we choose to start with happens to be idempotent, or, at the other extreme, it might require *N* operations. Proceeding in this way, Moore found a range for the number of required operations for each of the considered definitions:

$$\begin{aligned} \begin{array}{lll} &{} \quad \hbox {at least} &{}\quad \hbox {at most} \\ (W_1') &{}\quad 4N^3+2N^2 &{}\quad 6N^3 \\ (H_1') &{}\quad 4N^3+2N^2 &{}\quad 6N^3 \\ (H_2) &{}\quad 4N^3+2N^2 &{}\quad 6N^3 \\ (M'') &{}\quad 4N^3+3N-2 &{}\quad 4N^3+N^2+2N-2 \end{array} \end{aligned}$$

With regard to the infinite case, Moore made the slightly puzzling statement:

Here it is understood that $N, N^2, N^3$, and the symbols of addition and multiplication have a rather definite meaning, even if $N=\infty$, in connection with the notion of reading from the multiplication-table. (Moore 1902b, p. 491)

However, this last comment is to be interpreted, what was clear to Moore from his table in the finite case was that his ($M''$) would be the easiest definition to verify:

the definition ($M''$) seems to me to be an advantageous one both from the grouptheoretic [*sic*] and the logical standpoints. (Moore 1902b, p. 491)

Indeed, although we might question certain elements of Moore’s presentation (such as the above problem over the infinite case), his attempt at justifying his choice of definition was far more rigorous than that given by Huntington. Moreover, in contrast to Huntington’s sometimes extreme paring down of definitions, Moore sought a definition that, like our Definition 1, would lay bare the most important group properties.

### Variations on a theme: Huntington and Moore

Although, as we have just seen, Moore presented an approach to the study of group postulates that might be considered reasonably rigorous, it does not appear to have been taken up by his contemporaries—it is difficult to say why, although it may simply be that the rigour of his method was slightly undermined by the fact that it was still possible for aesthetics to colour any such considerations. Nevertheless, the study of group axioms continued, and the first person to respond to Moore’s approach, appropriately enough, was Huntington.

In his ‘Two definitions of an Abelian group by sets of independent postulates’ (Huntington 1903a), received by the AMS in October 1902 and published in January of the following year, Huntington did very much as his title indicates: he presented two definitions of Abelian groups, based on his earlier group definitions, with simplifications afforded by the presence of commutativity. For completeness, we record these definitions here, although we will not dwell on them (Huntington 1903a, pp. 27, 29):

[by three postulates] A set of elements in which a rule of combination $\circ$ is so defined as to satisfy the following three postulates shall be called an Abelian group with respect to $\circ$:

1. $a\circ b=b\circ a$, whenever *a*, *b* and $b\circ a$ belong to the set.
2. $(a\circ b)\circ c=a\circ (b\circ c)$, whenever $a, b, c, a\circ b, b\circ c$ and $a\circ (b\circ c)$ belong to the set.
3. For every two elements *a* and *b* ($a = b$ or $a \ne b$) there is an element *x* in the set such that $a\circ x = b$.

$$\ldots $$

[by four postulates] An Abelian group may be defined also by the following four postulates:

1$$'$$)
$a\circ b= b\circ a$, whenever $a, b, a\circ b$ and $b\circ a$ all belong to the set.
2$$'$$)
$(a\circ b)\circ c= a\circ (b\circ c)$, whenever $a, b, c, a\circ b, b\circ c, (a\circ b)\circ c$ and $a\circ (b\circ c)$ all belong to the set.
3$$'$$)
For every two elements *a* and *b* ($a = b$ or $a\ne b$) there is an element $x'$ in the set such that $(a\circ x')\circ b =b$.
4$$'$$)
If *a* and *b* belong to the set, then $a\circ b$ also belongs to the set.

To show that this second definition agrees with the first, we have only to notice that the truth of 3 follows at once from 2$'$, 3$'$, 4$'$. ($x =x'\circ b$.)

The reference above to Huntington’s further work as being a ‘response’ to that of Moore was perhaps an overstatement, for the style of Huntington’s paper is very much the same as that of his earlier articles. The only true influence from Moore comes in the following comment:

Professor Moore’s criticism of “multiple statements” suggested the present form of postulates 1 and 2. (Huntington 1903a, p. 27, footnote †)

The bulk of Huntington’s paper, however, is taken up by proofs of the independence of the respective sets of postulates. A postscript, added to the manuscript in November 1902, makes further reference to Moore’s work, but only in connection with independence: within the system labelled ($H_1'$) by Moore, either of postulates $2_1'$ or $2_2'$ may be deduced from the other three, resulting in a new system of axioms

$$\begin{aligned} \begin{array}{lll} (H_1'')&:&(2_2', 9', 10'), \end{array} \end{aligned}$$

‘in which the postulate $2_2'$ is “milder” than the postulate $2'$’ (Huntington 1903a, p. 30). Thus, despite his nod to Moore’s ideas about the simplification of statements, Huntington’s goals remained much the same as they had been before: the cutting down of the group definition as much as possible, with little regard given (unlike Moore) to the usability of the resulting definition—we might point, for example, to the persistence in his definition ($H_1''$) of peculiarities concerning closure. It might also be argued that an implicit conclusion of Moore’s work was that independence of postulates should be secondary to usability, but independence remained the focus of Huntington’s researches. That being said, however, Moore also continued to tinker with his definitions from the point of view of independence: a later short paper (Moore 1905) showed that ($3_r''$) is redundant in ($M''$), resulting in a streamlined system of postulates ($\bar{M}''$), to which Moore then added the commutative law to produce the system ($\bar{M}_A''$) for an Abelian group; he showed that the independence of the postulates in ($\bar{M}''$) carries over into ($\bar{M}_A''$).

Huntington’s paper of 1903 had already been in preparation when he became aware of the details of Moore’s analysis of group postulates (see Huntington 1903a, p. 30), so, if only for that reason, we should not condemn him for failing to adopt Moore’s ideas. In fact, Huntington’s subsequent publications suggest that he did in fact take Moore’s criticisms to heart; in a paper of the following year on the algebra of logic, he commented on the desirable form of postulates:

For the sake of elegance, every set of postulates should be free from redundancies; in other words, the postulates of every set should be independent, no one of them deducible from the rest. $\ldots$ Furthermore, each postulate should be as nearly as possible a simple statement, not decomposable into two or more parts; but the idea of a simple statement is a very elusive one, which has not yet been satisfactorily defined, much less attained. (Huntington 1904, p. 290)

A footnote attached at the end of this passage refers the reader to the comments on this matter in Moore (1902b).

A further, and longer, paper of 1905 saw Huntington perform a comprehensive analysis of axioms for various systems, including groups, in the style of Moore. Huntington developed a system of postulates for arithmetic in the real numbers, out of which he pulled a list of axioms defining an Abelian group (which, given the context, he now wrote additively). He also devoted space to the further study of group postulates in general:

The postulates for the theory of groups $\ldots$ carry the analysis farther, it is thought, than the earlier sets given by the writer and by E. H. Moore. (Huntington 1905a, p. 18)

In the interests of maintaining the flow of the narrative, we will not survey Huntington’s new analysis in all its details nor list his postulates here (see instead Appendix A): what is of interest to us now is the *style* of the work, for here Huntington abandoned his insistence on condensing the group definition into its most compact possible form and instead systematically developed sets of postulates numbering in double figures (another reason for relegating them to an appendix). Independence remained an important, but arguably secondary, concept,^50^ behind simplicity of the type espoused by Moore:

$\dots$ each postulate has been made as nearly a simple statement as seems possible. (Huntington 1905a, p. 34)

Huntington noted, however, that his postulate *G*9, an elaborate form of the associative law (see Appendix A.2), might yet be further subdivided, presumably in the manner previously hinted at by Moore for Huntington’s earlier associativity condition. On the whole, however, Huntington eschewed the extreme form of subdivision of postulates that Moore had advocated:

Most of the postulates embody, to be sure, a multitude of statements, corresponding to the multitude of elements in the group; but (except in case of *G*9) there seems to be no ground for distinguishing any of these statements from the rest; that is, there seems to be no ground for further subdivision of any of the postulates except *G*9. (Huntington 1905a, p. 34, footnote †)

This being said, Huntington nevertheless elected to retain the more complicated version of *G*9, perhaps because it fitted well into the flow of his presentation.

Thus, Huntington appears to have given some thought to Moore’s ideas concerning the simplification of postulates and to have arrived thereby at his own compromise.^51^ Despite a slight convergence of styles here, Huntington’s agenda remained very much his own, based perhaps on aesthetics, rather than the pedagogical considerations that seem to have entered into Moore’s thinking. We can perhaps see evidence of this by looking to a comment that follows Huntington’s statement of his postulate *A*1 (closure) for an Abelian group (see Appendix A.1):

This postulate states the fundamental property of a group $\ldots$ (Huntington 1905a, p. 22)^52^

However, when Huntington moved next to the more general setting of an arbitrary group, he reverted to what might be viewed as another rather over-elaborate treatment of closure: his postulate *G*1 asserts that if *a* is an element of the set in question, then so too is $a^2$, whilst his *G*2 requires that if *b* is a further element of the set, and $a\ne b$, then *ab* belongs to the set;^53^ the closure property in its standard, and simplest, form then appears as Huntington’s Theorem II, which of course requires no proof, being simply the amalgamation of *G*1 and *G*2. It may be that what we see here, as we saw in a similar setting for Moore, is a clash of the dual desires for simple postulates and simplicity of presentation.^54^

By 1905, this early twentieth-century study of the group postulates appears to have largely run its course: only one further paper by Huntington remains to be considered (Huntington 1905b). Indeed, the paper in question gives the impression that Huntington himself felt that there was little to gain from further tinkering with the groups axioms:

Further analysis of the postulates, such as I have attempted in [Huntington 1905a], does not seem likely to lead to practical advantage, except possibly in the case of the associative law. (Huntington 1905b, p. 183, footnote ‡)

Indeed, the phrasing of the associative law remained of interest to Huntington, and it is in this paper that we find the first explicit justification of his rather convoluted handling of both associativity and closure; in discussing his earlier investigations, Huntington noted that

$\ldots$ by a peculiar wording of the associative law, [the closure] postulate $\ldots$ could be made redundant. (Huntington 1905b, pp. 182–183)

Thus, Huntington had sought to exclude closure from his list of postulates, but perhaps not for the same reasons as we might exclude it today. Here, however, he acknowledged the drawbacks of doing so:

Since [the closure] postulate $\ldots$ is the fundamental postulate of the whole theory, the resulting definition [i.e., that which Moore labelled ($H_1$)] had obvious disadvantages. (Huntington 1905b, p. 183)

Huntington went on to refer to Moore’s versions of the postulates as ‘[m]ore convenient’ (Huntington 1905b, p. 183), and elsewhere in this second paper of 1905, we find him making comments that tally with those appearing in the Introduction of the present paper:

In §1 of the present note I propose a further modification of the Pierpont–Moore type of definition in the direction indicated by the latter part of Moore’s paper. Although the points of difference are so slight as to seem almost trivial, yet the introduction of postulate 4, demanding explicitly the uniqueness of the identical element, and the ‘weakened’ forms in which postulates 5 and 6 are now stated, will be found very convenient when one has to test a given system for the group property. (Huntington 1905b, p. 183)

The postulates listed by Huntington, as applied to a class *K* with a ‘rule of combination’, and including the 5 and 6 referred to in the above quotation, do indeed seem more usable that those in his earlier lists—note the reintroduction of the standard forms of closure and associativity (Huntington 1905b, p. 185):

Postulate 1. If *a* and *b* are elements of the class, then there is an element *c* in the class such that $ab= c$; and this element *c* is uniquely determined by *a* and *b*.

Postulate 2. The associative law holds throughout *K*; that is,

$$\begin{aligned} (ab)c=a(bc), \end{aligned}$$

whenever *a*, *b*, *c*, *ab*, *bc*, (*ab*)*c*, and *a*(*bc*) are elements of *K*.

Postulate 3. There is at least one element *i* such that $ii=i$.

Postulate 4. There is not more than one element *i* such that $ii = i$; that is, if *x* and *y* are elements such that $xx =x$ and $yy=y$, then $x =y$.

Postulate 5. If there is a unique element *i* such that $ii = i$, then either $ia =a$ for every element *a*, or else $ai =a$ for every element *a*.

Postulate 6. If there is a unique element *i* such that $ii=i$, then for every element *a* there is either an element $a_r$ such that $aa_r =i$, or else an element $a_l$ such that $a_la =i$.

Huntington’s first deduction from his definition is that the elements *i* of postulates 3, 4 and 5 are in fact one and the same and thus give a unique two-sided identity for the group. This last definition is therefore very close to our Definition 1, but with some small complications: the appearance of the redundant phrase ‘whenever $a, b, c, \ldots$’ in postulate 2, for example. In a later part of his paper, Huntington proved the independence of the above six postulates, whilst the final sections outline a rather different approach to the subject: the definition of a group by means a triadic relation (now more usually termed a *ternary* relation) on the underlying set. This was an approach suggested by Maxime Bôcher (1867–1918) in a lecture given in 1904. Neither Bôcher nor Huntington appear to have pursued this line of thought in any detail—perhaps because the necessary postulates are less transparent than those given above — although triadic relations later had a role to play in Huntington’s investigation of the postulates of other objects.^55^ I will therefore not explore this here, although a brief outline of this approach to groups is given in Appendix B.^56^

### New objects: J.-A. de Séguier and L. E. Dickson

That the heyday of the postulate analysis of groups was over by the middle of the first decade of the twentieth century can perhaps be seen from the comments of Huntington quoted in the preceding subsection: a great many alternative versions of the group definition had been derived and explored, but Huntington had arrived finally at something that was not too far away from our Definition 1, with the comment that further study was unlikely to ‘lead to practical advantage’. Before we can offer an assessment of the various postulational studies of the early twentieth century, it remains to deal briefly with some final contributions from the American school: two 1905 papers by Leonard Dickson that link back to the discussions of the Introduction and also look ahead to some of the later mathematics that postulate-analytic-type considerations may be said to have sparked.

Dickson was a former student of Moore who remained in Chicago after completing his PhD, so he would certainly have been aware of Moore’s work on group postulates, and probably also attended the seminar concerning Hilbert’s *Grundlagen*. Indeed, by 1905, he had already made contributions to the study of postulates for fields (Dickson 1903a) and algebras (Dickson 1903b). In 1905, he published two papers concerned with group postulates, but then—unlike Huntington, for whom postulational questions remained a major interest^57^—does not appear to have returned to this topic ever again.

The first of Dickson’s two papers of 1905 can in fact be dealt with very quickly, as it follows the pattern already established by Huntington and Moore: postulates are stated and compared with those of earlier definitions, simple consequences are deduced and independence of the postulates is established via the construction of appropriate counterexamples. There is little justificatory text, with the principal exception of the opening paragraph:

The simple definition here given for a general abstract group relates as to origin and character to Professor Moore’s two definitions [Moore 1902b]. A few days before the appearance of the addition [i.e., the Erratum] to his paper, Professor Moore remarked to me that one of his postulates relating to an inverse was redundant, meaning postulate ($3''_r$) of his second definition. I thought the reference was to his first definition and attempted to reconstruct the proof of a redundancy, there absent. This attempt led me to alter his postulate ($4_l$) to read $aa'_r = i_r$ instead of $a'_la = i_r$ and to note that postulate ($3_l$) becomes redundant in the altered set, thus obtaining the present definition. Subsequently I learned that Professor Moore, in his proof of the redundancy of ($3''_r$) in his second definition, had obtained relations [Moore 1905] sufficient to establish the present definition but had not applied them to set up the definition itself. (Dickson 1905a, p. 198)

With reference back to Moore’s postulates, we may easily unpick Dickson’s comments to see that the conditions set out in his definition of a group are simply closure,^58^ associativity, existence of a right identity and existence of right inverses with respect to that right identity: namely, the one-sided definition of a group that appears here as our Definition 2. Thus, we have Dickson to thank for a common undergraduate exercise (to show that Definitions 1 and 2 are equivalent) and also for the initial impetus for an algebraic line of enquiry that would take us beyond the bounds of group theory, so we do not discuss it here: see instead Appendix C.

The second of Dickson’s papers of 1905 is a little more interesting and had its origins in a book review that he had written the year before (Dickson 1904). This was a review of the first book to take *abstract* groups as its main focus: J.-A. de Séguier’s *Théorie des groupes finis: Éléments de la théorie des groupes abstraits* (de Séguier 1904).

De Séguier’s definition of a group was rather wordy, reminiscent in some ways of Cayley’s, in that the defining conditions do not appear as a clear numbered list, but are scattered across the text of one page (de Séguier 1904, p. 7). Nevertheless, de Séguier did mark out his postulates where they appeared, so Dickson was able to extract a definition from the former’s prose, via a new notion introduced by de Séguier (1904, p. 8):

The author introduces $\ldots$ a *semigroup* *G* in connection with any subset *S* containing a system of generators of *G*. The postulates defining *G* are: (1) associativity; (2) for any *a* in *S* and any *b* in *G*, there is at most one solution *x* in *G* of $ax = b$; (3) similarly for $xa = b$. (Dickson 1904, p. 160)

This definition is of course that given by earlier authors for a finite group, but applied to a set *G* that may be finite or infinite. Such a ‘semigroup’^59^ is clearly a group in the finite case, but of course not in the infinite case. As we know from earlier, such a ‘semigroup’ obeys the left and right cancellation laws, and de Séguier set out to prove this. However, Dickson took exception to a peculiarity in his phrasing:

The author states that it follows that $ax = ax'$ requires $x = x'$ for any *a* in *G*. If so, the definition itself might better read: for any two elements *a* and *b* in *G*, there is at most one solution *x* in *G* of $ax = b$. The conclusion was presumably reached about as follows: Express *a* in terms of the generators, say $a = a_1a_2a_3$. Then $a_1(a_2a_3x) = a_1(a_2a_3x')$ would require $a_2(a_3x) = a_2(a_3x')$, whence $a_3x=a_3x', x=x'$. However, this argument assumes that $a_2a_3x$ belongs to *G*. But it does not follow from the postulates that the product of every two elements of *G* belongs to $G \ldots$ (Dickson 1904, p. 160)

Dickson took issue with the fact that closure had not been demanded explicitly. One should probably assume that closure was being taken as an inherent property of the binary operation. However, as we have seen in the preceding sections, this is not a view that would have sat well with the postulate analysts, for whom closure was almost always required to be a postulate, or else an immediate and explicitly derived consequence of postulates. Indeed, further up the same page, Dickson had noted:

A group is defined by Huntington’s three independent postulates [Huntington 1901–1902a]; it being a theorem that the product of any two elements lies in the set. Most readers, I think, would find it more natural to have this property as a postulate, as is the case in Moore’s definitions. (Dickson 1904, p. 160)

He therefore suggested the explicit adjunction of the closure postulate to de Séguier’s definition of a ‘semigroup’. This done, he found a further fault with de Séguier’s treatment of groups, in which the transition from the finite case to the infinite did not run smoothly in all places. The problem concerns an ‘isomorphism’ between two groups *A* and *B*, which in this context means (in modern terms) a congruence *R* between the elements of *A* and *B* such that each $a\in A$ is *R*-related to at least one $b\in B$, and vice versa:

For two isomorphic finite groups *A* and *B*, the set $A_0$ of the elements of *A* which correspond to the identity of *B* form a group. For infinite groups the result is different and the author is in error. The converse statement $\ldots$ cannot be proved. I have constructed examples in which neither $A_0$ nor $B_0$ is a group. The correct theorem is that $A_0$ and $B_0$ are semi-groups. If either is a group the other is also, so that, for the ordinary case in which $B_0 = 1, A_0$ is a group. I will discuss this fundamental question in detail in the *Transactions*. (Dickson 1904, p. 162)

The *Transactions* paper referred to here is the second of the 1905 papers mentioned above, where Dickson did indeed address the question of ‘isomorphic’ groups, but with a slightly modified definition of ‘semigroup’: he replaced de Séguier’s second and third postulates, on solvability of equations, with the ‘equivalent but more desirable’ (Dickson 1905b, p. 205) left and right cancellation laws. He presented elaborate infinite examples in which $A_0$ and $B_0$ are not groups but ‘semigroups’, and, precisely in the manner of Huntington, demonstrated the independence of his postulates for a ‘semigroup’.

Although not at all semigroup-theoretic (in the modern sense), Dickson’s second paper of 1905 stands at the beginning of the development of the modern theory of semigroups, having at least provided the English-reading mathematical world with the word ‘semigroup’. Indeed, the paper appears to have been cited much more often in this capacity than in connection with its broader content, perhaps because the problem that it treats is a rather minor one. Taken together, however, Dickson’s two postulate-analytic papers of 1905 have the additional significance that they appear to mark the end of the often feverish investigation of group axioms that occupied several American mathematicians in the early years of the twentieth century.

## Further contributions

Although the focus of this paper is the postulate analysis carried out in the USA during the first decade of the twentieth century, it is instructive for us to look beyond this period. The studies that we saw in the preceding section had largely petered out by about 1910, although we do still see glimpses of postulational work by American authors in the decades that followed, some of which will be indicated briefly here. Some postulational work was also carried out, apparently independently of the American work that had come before, by a small number of German mathematicians, mainly during the 1930s, so I also include some brief notes on this here, with a view to expanding on this in the future. Finally, a rather more subtle strand of postulate-type work may also be found in some publications by Russian authors—again, I give a short account of this here, but hope to deal with it at greater length elsewhere.^60^

### Later American work

The postulate analysis that we have studied in detail here has been confined to a single topic (namely groups) and to a short period (the first decade of the twentieth century), but these investigations continued beyond our chosen timeframe, and considered many objects other than groups. Dickson, for example, handled the axiomatics of fields (Dickson 1903a, 1905a) and of linear associative algebras (Dickson 1903b). Moore’s postulational studies, on the other hand, were confined to groups, as far as algebra was concerned, although he later attempted to apply the postulational approach to analysis (see Siegmund-Schultze 1998). But of the main characters to have appeared in the present paper, it was Huntington who remained prominent in the wider postulational studies; Bell later referred to him as ‘the most prolific contributor to postulational analysis’ (Bell 1938, p. 18). Indeed, his list of publications bears this out, for we find there postulational studies of the real numbers (Huntington 1903c, 1905a) and complex numbers (Huntington 1905c), of abstract fields (Huntington 1903b), of the algebra of logic (Huntington 1904, 1933), and of assorted other axiomatically defined objects (Huntington 1902a, b, 1906, 1916, 1924a, b, 1927, 1935).

As the decades passed, the stream of papers on postulate analysis slowed, but did not dry up completely: beyond Huntington’s extensive efforts, we may find examples of postulational work in almost every decade of the twentieth century. Hurwitz (1907), for example, was directly inspired by Huntington to study the axiomatics of Abelian groups, and over half a century later, Harary (1961) was still referring back to Huntington when he considered the question of what independence of postulates ought to mean. Fields saw further postulational study at the hands of Wiener (1920), whilst Boolean algebras also proved to be popular objects of study.^61^ Groups continued to receive attention, with, for example, a range of attempts to characterise them in terms of an operation of division,^62^ or non-associative operations more generally (Bernstein 1938).

It might be noted from the references given in the preceding paragraph that the 1930s seem to have seen a renewed interest in postulational questions, after something of a slump during the preceding two decades. We could put this down to the growing interest in abstract algebra following the publication of van der Waerden’s seminal text *Moderne Algebra* (1930–1931), but, in the American context, we can perhaps also cite the popularisation work of Bell: during this decade, Bell was promoting abstract algebra and the postulational method not only to a general readership, but also to mathematicians (see Hollings 2016); for example, a paper entitled ‘Unique decomposition’ (Bell 1930) provided a rather appealing abstract framework for the study of a range of algebraic objects. Although, as I argued in Hollings (2016), Bell’s own contributions in this direction were rather limited, he seems to have been encouraging his students to consider postulational questions. Thus, for example, Morgan Ward, who completed a PhD at Caltech under Bell’s supervision in 1928 considered the postulates necessary to produce an abstract system in which unique factorisation is possible (see Hollings 2014b, §4.3). Whilst pursuing a related problem, another of Bell’s students, A. H. Clifford, provided a study of the independence of his derived postulates—not because this was the main thrust of his work, but perhaps because (with some prompting from Bell?) it seemed like a natural thing to do (see Hollings 2014b, §4.5). And, indeed, by this stage, most postulational treatments that were appearing in print were of this nature: incidental parts of much broader studies, rather than investigations for their own sake.

We conclude this subsection with a few comments on a postulational study that, like Ward’s, had an arithmetical starting point, but was rather broader in its intended scope: H. S. Vandiver’s development (later co-authored with his student M. W. Weaver)^63^ of various number-theoretic concepts from the standpoint of abstract algebra. Whereas the question of divisibility, leading to unique factorisation, was the key idea for Ward’s scheme, Vandiver’s goal was to derive a single over-arching abstract theory for the positive integers, of which prime factorisation would only be a part. I do not attempt to give a treatment here of the work of Vandiver and Weaver, but note that it is an interesting strand of research that would bear further investigation, not least because it appears (based, admittedly, only on a superficial impression) to have certain parallels with nineteenth century British contributions to symbolic algebra. As with other topics alluded to in the present section, I hope to deal with the work of Vandiver and Weaver in a future paper.

### Postulate analysis in other countries

The pursuit of postulational work in the USA during the first decade of the twentieth century appears to have had a clear overall theme: the focus was on the search for minimal (whatever that might mean) sets of independent postulates for given objects. Even in much of the later work cited in the preceding subsection, this remained the major preoccupation. As indicated at the beginning of this section, investigations akin to the American postulate analysis appeared in the work of mathematicians of other nations. However, the overall ‘shape’ of these studies remains unclear, as does their dependence, or otherwise, on the American work that had come before—further investigation is needed. In the meantime, I give a rough indication of some topics that I believe it would be interesting to study in greater depth.

#### Germany

Although perhaps not carried out on quite the same scale as work in the USA, some postulate-related investigations appeared in the German literature, particularly in the 1930s.^64^ As in the American case, some impetus may have been provided here by the publication of van der Waerden’s *Moderne Algebra* in 1930/1931, although the latter is perhaps more important in this context for the clarity it brought to the teaching of algebra.

One German mathematician who was particularly systematic in postulational work was Fritz Klein-Barmen (a.k.a. Fritz Klein),^65^ who studied axiomatically defined systems, usually with symmetric pairs of binary operations (‘Verknüpfungen’). Since his inspiration came from considerations in logic, Klein-Barmen’s early interest was in systems whose pairs of operations satisfy a distributive law (Klein 1929). Thus, Klein-Barmen’s investigations were of a rather different character from the postulate work that we have seen earlier in this paper; rather than simply seeking new systems of axioms for known objects, his experimentation with postulates led him to new algebraic constructs, some of which have subsequently seen further study: his *Verband* (Klein 1932) is a lattice,^66^ whilst his *A*-*Menge* (Klein 1931) is a distributive lattice. Klein-Barmen also considered systems with a single binary operation; his *B*-*Menge* (Klein-Barmen 1933, §3), for instance, is a special case of a semilattice. As such, it was identified by A. H. Clifford as having links to his own work on factorisation in semigroups (see Hollings 2014b, §4.5). The bulk of Klein-Barmen’s later work appears to have retained the axiomatic flavour: a decade later, for example, he worked on the axiomatics of lattices (Klein-Barmen 1933), and of what came to be known as skew lattices (Klein-Barmen 1940); a little later, he turned to systems of axioms for various classes of semigroups (see, for example, Klein-Barmen 1956, 1958).

Also from the 1930s, we mention the systematic axiomatic work of Reinhold Baer and F. W. Levi in their 1932 paper ‘Vollständige irreduzibele Systeme von Gruppenaxiomen’.^67^ As its title suggests, this paper of Baer and Levi contains a study of various systems of group-like axioms, drawing not only upon those previously written down by Weber (1882), but also upon the textbook definitions of groups presented by Hasse (1926) and Loewy (1910, 1915). The key observation in Baer and Levi’s paper is that if we are given a composition

2 $$\begin{aligned} a=b\cdot c, \end{aligned}$$

then the group axioms may be expressed by two basic postulates:

(I) in (2), each element is uniquely determined by the others;
(II) the composition is associative.

Baer and Levi noted further that postulate (I) may be subdivided into six independent statements, three for existence and three for uniqueness. Thus, we may choose to define a group by the following seven independent axioms, where ‘${\mathfrak {E}}$’ stands for ‘Existenz’, and ‘${\mathfrak {U}}$’ for ‘Unität’:

($${\mathfrak {E}}_a$$)
for any two elements *b*, *c*, there exists at least one *a* satisfying (2);
($${\mathfrak {E}}_b$$)
for any two elements *c*, *a*, there exists at least one *b* satisfying (2);
($${\mathfrak {E}}_c$$)
for any two elements *a*, *b*, there exists at least one *c* satisfying (2);
($${\mathfrak {U}}_a$$)
for any two elements *b*, *c*, there exists at most one *a* satisfying (2);
($${\mathfrak {U}}_b$$)
for any two elements *c*, *a*, there exists at most one *b* satisfying (2);
($${\mathfrak {U}}_c$$)
for any two elements *a*, *b*, there exists at most one *c* satisfying (2);
($${\mathfrak {A}}$$)
the associative law holds.

Baer and Levi chose not to express associativity in terms of existence and uniqueness, but noted that this had been done by Huntington—as we saw in the ‘existence’ parts of the various forms of Huntington’s versions of the associative law in Sects. 4.2 and 4.3. Like most authors, however, Baer and Levi took the existence of all such products to be a part of their definition of composition, so there was no need for them to separate out the ‘existence’ and ‘uniqueness’ parts of associativity.

It is clear that Baer and Levi’s conditions (${\mathfrak {E}}_a$) and (${\mathfrak {U}}_a$) ensure that the binary operation is well-defined. Notice that their (${\mathfrak {E}}_b$) and (${\mathfrak {E}}_c$) are simply the ‘solvability’ conditions that we have seen in earlier sections: the former ensures that the equation $a=xc$ is solvable for any *a* and *c*, whilst the latter relates to the solvability of the equation $a=by$ for *y*. The corresponding ‘${\mathfrak {U}}$-postulates’ provide uniqueness of the solutions; alternatively, these may be interpreted, respectively, as the right and left cancellation laws. In the terminology found, for example, in Clifford and Preston (1961, §1.11), a semigroup that satisfies (${\mathfrak {E}}_b$) (with a slight modification—see Appendix C) is said to be *left simple*, whilst a semigroup with (${\mathfrak {E}}_c$) (again with a small modification) is called *right simple*.^68^ Thus, Baer and Levi’s paper is essentially a study of the interactions between the properties of left and right cancellation and those of left and right simplicity; some other studies of combinations of these properties are outlined in Appendix C.

The principal goal of Baer and Levi’s paper was to find a system of axioms for a group that is both *complete* (*vollständig*) and *irreducible* (*irreduzibel*), where a set of axioms is *complete* if it defines a group, and *irreducible* if the axioms therein are independent. As a notational convenience, the first six postulates in the above list were written by Baer and Levi in the form of a matrix:

3 $$\begin{aligned} \begin{pmatrix} {\mathfrak {E}}_a &{}\quad {\mathfrak {E}}_b &{}\quad {\mathfrak {E}}_c \\ {\mathfrak {U}}_a &{}\quad {\mathfrak {U}}_b &{}\quad {\mathfrak {U}}_c \end{pmatrix}. \end{aligned}$$

They proved the following result (Baer and Levi 1932, §§2–3):

##### Theorem 1

A system $\varSigma$ of group axioms is complete if and only if the following conditions hold:

1. $$\varSigma $$ contains $$({\mathfrak {A}})$$;
2. $\varSigma$ contains at least one axiom from each row and each column of (3);
3. $\varSigma$ contains two axioms of the first row of (3);
4. if $\varSigma$ does not contain both $({\mathfrak {E}}_b)$ and $({\mathfrak {E}}_c)$, then it contains both $({\mathfrak {U}}_b)$ and $({\mathfrak {U}}_c)$.

Moreover, the conditions (1)–(4) are irreducible.

At the end of the paper, Baer and Levi provided a brief investigation of what happens in the case of a finite group. Thus, once their axioms are unpicked, we see that Baer and Levi’s studies were not too far away from the spirit of American postulate analysis.

For other German work on a postulational theme, we may look to the paper Lorenzen (1944), which collected and organised a great range of group properties, listing more than 40 in all, from which Lorenzen then derived 20 different systems of 3 or 4 axioms, each defining a group. His approach was very much in the spirit of the work of Huntington and others, but no reference was made to these earlier investigations, only to the paper of Baer and Levi that we described above, and to the 1935 study by Garver that was mentioned in passing in the preceding subsection. Elsewhere (Lorenzen 1940), Lorenzen introduced (in a paper of just one page) an axiomatisation of groups in terms of an operation of division, something that was also done earlier by some American mathematicians, as we noted in Sect. 5.1. Lorenzen’s investigations, in conjunction with those of Baer and Levi, later gave rise to a similar study in a broader context: the thesis Stolt (1953) and papers Stolt (1955a, b, c, 1956a) catalogue a range of properties for semigroups, from which are extracted sets of postulates that may be used to define groups.

#### Russia and the USSR

A point that I will return to in Sect. 6 is that the vast majority of textbook definitions, and the greater proportion of monograph definitions, of a group follow our Definition 1, rather than our Definition 2. However, one particularly noteworthy instance of the latter being presented as the starting definition of a group is that found in Otto Schmidt’s 1916 Russian monograph 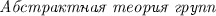 (*Abstract theory of groups*). Just as de Séguier’s text of 1904 is regarded as the first book to give an entirely abstract treatment of groups in the finite case, Schmidt’s was the first to handle *infinite* groups abstractly.^69^ The first chapter opens with a brief introduction to the notion of composition or ‘symbolic multiplication’ (‘
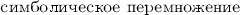
’) of elements of an aggregate ${\mathfrak {G}}$, which leads into the following definition:^70^

A similar aggregate ${\mathfrak {G}}$ is called a *group* if it satisfies the following conditions.

1. *The product of any two elements of* ${\mathfrak {G}}$ *must itself belong to* ${\mathfrak {G}}$.
2. *Symbolic multiplication must be subject to the combinative (associative) law, i.e., for any three elements of* ${\mathfrak {G}}$
  $$\begin{aligned} A(BC)=(AB)C. \end{aligned}$$
3. *In the aggregate* ${\mathfrak {G}}$ *there must be at least one element* *I* *possessing the property that for every element* *A* *from* ${\mathfrak {G}}$ *we have the equality:*
  $$\begin{aligned} AI=A. \end{aligned}$$
4. *For a particular choice from the elements* *I* *and for every* *A* *from* ${\mathfrak {G}}$ *there must exist an element* *X* *in* ${\mathfrak {G}}$ *such that*
  $$\begin{aligned} AX=I. \end{aligned}$$

Schmidt offered no explanation as to why he chose to adopt the one-sided definition of a group, but, of course, one of his first results is that the one-sided identity, as well as being unique, is in fact two-sided, and the same for inverses. He must simply have preferred that presentation.^71^ Schmidt provided no general discussion of postulates in the manner that we have seen in earlier sections, but it is reasonable to suppose that this was of little relevance to him (as indeed it is for most group-theoretic texts—see the comments in Sect. 7): for his purposes, the group concept was an established one that simply needed to be introduced to his readers.

Schmidt’s monograph, together with his algebraic work more generally, marked the beginning of an interest in abstract group theory in Russia.^72^ His book almost invariably appears in the bibliographies of subsequent Russian books on group theory; we might cite, for example, Schmidt’s student A. G. Kurosh: the preface of his 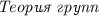 (*Theory of groups*) explicitly credits Schmidt with the creation of the Soviet school of group theory (see, for example, Kurosh 1967, p. 9).

One book that is relevant for our present purposes and that might best be described as an off-shoot from the group-theoretic tradition initiated by Schmidt is A. K. Sushkevich’s 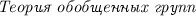 (*Theory of generalised groups*) of 1937. As its title suggests, this monograph concerns various generalisations of the group concept: mostly semigroups,^73^ with some material on quasigroups, but also a final chapter on assorted other systems. However, Sushkevich saw his text as being embedded in the existing group-theoretic literature:

Besides an acquaintance with general mathematical culture, the reading of this book requires simply a familiarity with the classical theory of ordinary groups, at least that found in Academician O. Yu. Schmidt’s book “Abstract theory of groups”.^74^

Sushkevich’s focus on semigroups stemmed from the fact that his primary motivation was the study of not-necessarily invertible transformations of sets, closed systems of which of course form semigroups. Nevertheless, he also provided some discussion of postulates: beginning on his p. 39, he undertook an ‘Analysis of the general laws of operation’ (‘
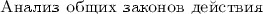
’), which took in many of the postulates that we have seen in earlier sections. Indeed, Huntington, Moore and Dickson are all cited briefly in connection with the group definitions that they put forward, independence of postulates is considered, and there is even a brief nod to the notions of ‘disjunctive’ and ‘categorical’ sets of postulates.^75^ A significant portion of Sushkevich’s discussion of postulates is taken up by the study of certain weakened versions of the associative law, some of which he had previously employed in a paper (Suschkewitsch 1929) now regarded as one of the first on quasigroups (see Pflugfelder 2000). However, despite Sushkevich’s lifelong interest in the history of mathematics,^76^ the *historical* treatment of postulate analysis in *Theory of generalised groups* is somewhat terse: for a ‘Literary-historical survey’ (‘
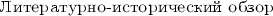
’) of this topic, we must turn instead to Sushkevich’s doctoral dissertation (Sushkevich 1922), written around 1918, of which *Theory of generalised groups* is in essence a much-expanded version. Sushkevich’s survey takes up roughly one quarter of the whole dissertation,^77^ and covers some of the same ground as our earlier sections. However, I save detailed discussion of this for a further paper. Although I have argued elsewhere (Hollings 2014b, §3.4) that Sushkevich’s impact in research mathematics was minimal, his approach to the group definition is nevertheless of interest for the treatment that he gave it in his teaching materials—he was a noted pedagogue and author of several textbooks on algebra, some of which present students with basic analyses of the relevant postulates—but this too I save for a later paper.

## Later points of view

We provide here an indication of later opinions, both positive and negative, of the value of postulate analysis. We begin in Sect. 6.1 with views of algebra and of postulate analysis presented by mathematicians who were mostly^78^ approaching the subject from the research point of view; in Sect. 6.2, we return to an issue that was discussed briefly at the beginning of the paper: the comparative pedagogical merits of different group definitions, and of the postulational method more generally.

### Research mathematics

Having begun this article with a quotation from E. T. Bell, let us now consider some further comments of his on the subject of postulate analysis. At a meeting marking the fiftieth anniversary of the AMS, Bell gave an address on the contributions of American mathematicians to abstract algebra (subsequently published as Bell 1938). Given his enthusiasm for the postulational method, and his extensive promotion of it (Hollings 2016), it is no surprise that this features quite prominently:

It may be said that American work in this general field has been sharper and clearer on the whole than that done elsewhere. (Bell 1938, p. 19)

But this is not just Bell’s bias showing through: postulate analysis also received special mention in a lecture by G. D. Birkhoff at the same meeting (Birkhoff 1938, pp. 280–281). What both appeared to have agreed upon is that postulate analysis was a specifically *American* contribution to mathematics;^79^ their willingness to ‘claim’ it for the USA speaks to a belief in its value. Similar hints of approval may be seen in the remark of Easton, quoted in the Introduction to the present paper. However, as we move further away from the first decade of the twentieth century, enthusiasm for postulate analysis seems to wane; for example, it received only fleeting mention in two later survey volumes of American mathematics: Tarwater (1977) and Duren (1989). Views as to what is important appear to have changed.^80^

We note that even Bell’s view of postulate analysis was not an unqualified one. Some years later, he condemned ‘the decadent vice of playing with barren postulates’ (Bell 1952, pp. 27–28), and in his 1938 article, we find hints that he was aware of the trend mentioned in Sect. 5.1 of the present paper—that by the 1930s, postulate analyses were more often appearing as small parts of larger works, rather than as ends in themselves:

An inspection of the current literature shows no decline in the application of the postulational method. Much of the contemporary work has an ulterior motive, apparently, in topology or elsewhere $\ldots$ (Bell 1938, p. 19)

That there ought to be a reason for choosing particular postulates was something that Bell had noted at the end of the preceding decade in his monograph *Algebraic arithmetic*:

What is wanted $\ldots$ is a workable set of postulates which will reveal at a glance those properties of the elements considered which are of mathematical importance and which it is desirable to abstract $\ldots$ (Bell 1927b, p. 59)

And the 1938 article again expands on this theme:

But unless there are available a few suggestive algebraic phenomena in the beginning, what is to determine the direction of abstractness and generality? Possibly no determination is required; but if such is the fact, there exists no evidence in support of it. Why, in particular, should some algebraist prefer to elaborate one set of postulates rather than another? For at least half a century before the intensive study of groups began, it would have been easy for any logician to state the postulates for a group and to get what superficialities he could out of them; but it was only when a considerable body of detailed information about particular groups and their applications was available that rapid progress was made in the algebra of abstract groups. (Bell 1938, p. 5)

Other comments by Bell are, however, harder to agree with. For instance, he seized upon a comment by J. H. C. Whitehead (1938, p. 495) that ‘[t]he systematic study of abstract groups may be said to have been originated by L. E. Dickson’ and concluded that Whitehead must have been referring to Dickson’s postulational work; he even went so far as to offer a correction: that Moore and Huntington ought also to have been credited in this connection. Bell noted further:

The interest here of this early work of Huntington, Moore, and Dickson is the impulse it imparted to abstract algebra in America. (Bell 1938, p. 17)

This is certainly true within Bell’s analysis, but the above-cited survey volumes do not present a good case for the influence of postulate analysis. One retrospective account certainly places Dickson at the beginning of the development of abstract algebra in America, but not for his postulational work (Fenster 2007)—we will return to Dickson’s wider group-theoretic writings in Sect. 7. Another account, by Garrett Birkhoff, however, seems to be broadly in agreement with Bell, although it is critical of the latter’s ‘colorful’ treatment of the subject (Birkhoff 1973, p. 764). In Birkhoff’s account, we find sound-bites worthy of Bell, with a comment, for example, on the ‘[t]he emancipation of algebra from exclusive concern with the real and complex fields’ (Birkhoff 1973, p. 763); we also find the following in reference to the work of Huntington and Moore:

Partly as a result of such papers, the postulational approach to algebra finally became standard $\ldots$ (Birkhoff 1973, p. 764)

However, it is possible that Birkhoff gave a further indication of his views in the heading of the sixth section of his article: ‘Some deeper developments’. A distinction from postulate analysis was apparently being drawn here, with the implication that the latter is not ‘deep’.^81^

Returning to the 1930s, we find other comments on the nature of abstract algebra and the postulational method in an article by Saunders Mac Lane on recent advances in algebra (Mac Lane 1939). In a section headed ‘What is algebra?’, Mac Lane provided the following definition:

Algebra concerns itself with the postulational description of certain systems of elements in which some or all of the four rational operations are possible: fields, linear algebras, Lie algebras, groups. (Mac Lane 1939, p. 17)

However, he cautioned that

[t]he abstract or postulational development of these systems must then be supplemented by an investigation of their “structure”. (Mac Lane 1939, pp. 17–18)

Mac Lane explained that by the latter he meant the study of substructures and their interrelations, of automorphisms, of simple structures, and so on, thus giving a more rounded view of algebra than his initial comments on the postulational method may suggest. He concluded by offering the following further characterisation of algebra:

*Algebra tends to the study of the explicit structure of postulationally defined systems closed with respect to one or more rational operations.* (Mac Lane 1939, p. 18, Mac Lane’s italics)

Thus, the postulational method appeared now as a scheme for describing algebraic structures, rather than as an end in its own right. Mac Lane noted, however, that this description does not easily encompass, for instance, topological approaches to algebra:

As with many hyper-generalizations, our statements fit the facts only when the facts are first slightly distorted! (Mac Lane 1939, p. 18)

Nevertheless, Mac Lane had arrived at a definition of algebra that perhaps tallies more with modern views than with that offered by Bell.

### Pedagogy

Turning now to pedagogical considerations, we note that when undergraduates meet groups, they are often presented, in the first instance, with a list of axioms, which are then justified by appeal to natural examples of groups, symmetric groups in particular. The definition that students most often encounter during introductory courses on this topic is our Definition 1. This is probably because, as I argued in the Introduction (and also to some extent in Sect. 3), this is the definition that clearly encapsulates the most relevant properties of groups; as Bell would have it, this definition ‘reveal[s] at a glance those properties of the elements considered which are of mathematical importance and which it is desirable to abstract’ (see Sect. 6.1).^82^ It is probably for this reason that the vast majority of books (textbooks in particular) include this as their standard definition of a group: of 72 books sampled by the author,^83^ 50 (69%) started from our Definition 1 (possibly without an explicit demand for closure^84^). The remaining 22 books employed a variety of slightly different definitions, a few in terms of quotient operations, for example, or (for those that dealt exclusively with finite groups) cancellation. A couple opt for something rather different, although their reason for doing so is not always clear, given that the deduction of the ‘usual’ group properties invariably follows immediately after the definition.^85^ Of the 72, only 9 (12.5%) give the one-sided definition.^86^ If we restrict our attention only to the textbooks in the sample (47 out of 72—although it is sometimes difficult to draw the line between textbooks and monographs), 32 (68%) employ Definition 1; all 9 of the books that use the one-sided definition come under the heading of ‘textbooks’ (and hence represent 19% of textbooks in the sample). This rough analysis of the group definitions found in a range of books serves to confirm the popularity of Definition 1, but throws up the question of why a significant proportion of textbook authors elect to start from the one-sided definition. Little indication is given in the books, so we are left to speculate that the authors were, like Huntington and others, motivated by aesthetics, or that they felt that it would be a useful for the reader to see the deduction that the identity and inverses in a group are in fact two-sided, thereby gaining further insight into what a group is via a little light postulational work.

The introduction of groups (or other objects) via postulates can of course lead to the misapprehension amongst students that group theory and group axiomatics are one and the same.^87^ At the same time, a very abstract axiomatic approach will not be well suited to all learners. Indeed, around the time that Bell was extolling the virtues of the postulational method, and Mac Lane was indicating that abstract algebra is more than just postulates, a modest literature was developing on the use of postulates in the learning of algebra. We point, for example, towards Weiss (1939) on the choice of subject matter for algebra courses within a crowded US liberal arts curriculum. Weiss remarked that ‘[i]n recent years algebra has been dominated by the axiomatic point of view’ (Weiss 1939, p. 635) and proceeded to cite the definitions of algebra given by a number of well-known mathematical authors, including that of Mac Lane, quoted above. All those referenced were broadly in agreement that the postulational method lies at the foundation of algebra, and so Weiss’ conclusion was that:

This point of view, then, I believe we may take as our guide for an introduction to algebra; for although, as is well known, algebra makes use of ideas far afield from those embodied in the closure of a set under rational operations, the beginnings of the subject lie in this concept. (Weiss 1939, p. 636)

Nevertheless, Weiss’ suggested approach was pragmatic, rather than formalist:

Although I propose that an introduction to $\ldots$ abstract concepts $\ldots$ should form the motif of the course, I do not believe that a course for an undergraduate can begin with their satisfying axiomatic development, for the usual undergraduate has not sufficient background to make these concepts real to himself. Before these abstract concepts can be given to the student, material must be at hand so that he can see the need for such generalization and abstraction. To demand commutivity of an operation seems meaningless if the student has not had experience with non-commutative operations; to prohibit the presence of zero divisors in a set seems extraneous if the student has not had examples of sets in which zero divisors occur naturally. (Weiss 1939, pp. 636–637)

Weiss went on to explain how the introduction of ‘concrete’ concepts such as polynomials might be recast in a manner that lends itself to their later abstract treatment and thus provide students with an idea of what to postulate in a given situation.

Of relevance for our present purposes is Weiss’ discussion of groups in this context:

A first question in discussing the group is of course how redundant to make the set of postulates. For the sake of pedagogy, I have always given the postulates in what might be called super-redundant form. After demanding the closure of the set and the associative law for the law of combination, I have demanded the existence of a commutative identity and of a commutative inverse for each element of the set. (Weiss 1939, p. 639)

Weiss attached some value to the postulational method:

If training in postulate work is wanted, these postulates may of course be weakened and the student left to prove these fundamental properties of the identity and inverse. (Weiss 1939, p. 639)

It is not explained whether the ‘training in postulate work’ would be intended for its own sake, or simply as a way of helping the student to assimilate the group concept; in either case, the benefit to the student is clear.

Weiss’ article is couched in very general terms, being, in essence, an argument for the algebra teaching in US liberal arts colleges to be brought up to the same standard as that of calculus and analysis. A rather more detailed paper, from just a few years later, is that of Stabler (1943), entitled ‘Boolean algebra as an introduction to postulational method’. Recall from Sect. 5.1 that the postulates for Boolean algebras had by this time received much attention from the postulate analysts; these investigations were the starting point for Stabler’s article, which begins:

The purpose of this paper is to suggest how a simple system of postulates and theorems for Boolean algebras might be used as an introduction to postulational methods in an elementary course in foundations, or fundamental concepts, of mathematics. (Stabler 1943, p. 106)

Stabler’s reasons for believing that such an introduction is desirable appear to mirror those of the original postulate analysts—that the study of postulates has a genuine benefit for mathematical understanding:

Postulational theory often is introduced in such a course by the study of significant sets of postulates closely related to elementary algebra or geometry. This procedure undoubtedly is valuable. The approach suggested here, however, begins with postulates for a mathematical system quite different from the ordinary systems of elementary mathematics. (Stabler 1943, p. 106)

In contrast to the earlier researchers, however, his approach placed emphasis on postulates that are easily grasped:

At the same time, the postulates are so selected that only one of them would seem unnatural or queer to undergraduates. It is believed that there may be some psychological advantages in an approach of this kind. (Stabler 1943, p. 106)

Having given his postulates for a Boolean algebra, Stabler next outlined brief arguments for their consistency and deduced their basic consequences, before giving examples of concrete interpretations, and considering the ‘categoricalness’ of his system.^88^ Stabler’s conclusions about what his article had achieved provide a nice summary of what he believed to be the benefits of postulate analysis:

The postulational approach to Boolean algebra suggested here seems to have certain possibilities for introducing postulational methods and concepts to persons with little previous knowledge of foundations of mathematics or abstract mathematical systems. Thus, it furnishes a simple approach to the study of consistency, independence, and categoricalness of postulates; and it gives practice in easy formal deduction under novel conditions. At the same time, it serves to emphasize the relative nature of the truth of the theorems in any mathematical system. Finally, by examining two equivalent but quite dissimilar sets of postulates, it suggests that there is considerable freedom of choice as to the undefined concepts and fundamental propositions of such a system. (Stabler 1943, p. 110)

Although usually unstated in the papers that we have considered here, I believe that the above quotation provides a sound representation of the attitudes of most of the early postulate analysts.

So far, we have cited positive comments on postulate analysis from the second half of the twentieth century, and we have observed its neglect in survey volumes from the 1970s and 1980s. In the interests of balance, we should also note that rather more negative opinions have also been expressed in print, particularly in the educational setting. Denbow (1955, p. 234), for instance, decried

[t]he extreme form of “postulationism” [which] can lead to an educationally disastrous breakdown of communication between specialist and layman.

Denbow was writing in particular reference to adult education and argued that the use of postulates rejects an evidence-based view of mathematics. Similar comments were made a few years later in criticism of the ‘New Math’ movement in the USA (and elsewhere).^89^ In this connection, we might also make brief reference to criticisms of the style of mathematics advocated by Bourbaki.^90^ In the interests of saving space, and to retain the focus on the original postulate analysts, I have deliberately sidestepped both ‘New Math’ and Bourbaki in the present paper. For the latter, axiomatic formalism was a general organising principle, rather than a method of gaining understanding, but a comparison of the two parallel approaches might yet be made.^91^

## Conclusion

The postulate analysis that was carried out by American mathematicians in the first decade of the twentieth century was, in some cases, propelled by aesthetic considerations, or by a desire for greater precision in presentation, but more often, as I have tried to argue, by the genuine belief that the deconstruction of the postulates for a group, or any other structure, was an effective way of gaining an understanding of that concept. More generally, the pursuit of postulate analysis perhaps has a parallel with an argument that is often made in the defence of school mathematics teaching: that it is not necessarily the *content* of lessons that matters in all cases, but the training that the learning of mathematics provides in particular ways of thinking. In a much narrower context, it might be argued that the study of postulate analysis provides training in a particularly logical way of thinking (as argued by Stabler in Sect. 6.2) and that the specific content of the postulates under consideration does not matter.

Let us return to the theme of Sect. 6 by considering the ways in which postulate analysis manifested itself in the major group theory texts of the early twentieth century. Zassenhaus (1937), for example, said nothing on the subject, nor did van der Waerden (1930);^92^ a little earlier, Miller *et al.* (1916, p 85) had made a nod to Huntington’s postulate work, but only in a historical note, and with no substantial indication of the method. Indeed, the same may be said of Speiser (1923, p. 12). Carmichael (1937, p. 395) touched briefly upon the postulational method by noting ‘certain redundancies’ and cutting his group definition down to the one-sided version, but made no reference to the postulate analysts.^93^ Much closer in time to the heyday of postulate analysis, Burnside (1897, 2nd ed., pp. 452, 464) had cited Moore—but only in connection with his wider group-theoretic work; Huntington was not mentioned at all. Indeed, Huntington’s appearances in group theory texts are rare; Moore seems to be cited much more often—but, again, always for work other than postulate analysis.^94^ Barnett (2016, p. 11) notes this as a feature common to the postulate analysts: if they are remembered today, then it is typically for work in other areas.^95^

In this latter connection, let us consider the legacy of Leonard Dickson. Contrary to the interpretations of E. T. Bell that were quoted in Sect. 6.1, whenever Dickson is cited in connection with group theory, it is not for his postulational work, but often for his text on linear groups (Dickson 1901). Dickson’s book contains no references to postulational methods, which is perhaps unsurprising, since it pre-dates the major American works in the direction. What is striking, however, is the fact that Dickson listed no group postulates at all; his only definition of a group was, following Jordan (1870), that of a general linear homogeneous group (Dickson 1901, pp. 75–77). The general concept of a group does not appear explicitly, although Dickson seems to have expected his readers to know what this was.^96^ Perhaps we need to revise the date in the Bell quotation with which we began this paper? Perhaps in 1901 everyone already knew what a group was? And yet Dickson readily contributed to the postulate analysis that emerged in the few years following the publication of his book. He appears to have seen some value in it. I argue that in his 1901 text he did not need the general notion of a group—the specific cases handled there were enough—but that, along with the other postulate analysts, he was nonetheless interested in gaining a greater understanding of the group concept via the study of its postulates, in the manner described by Bell.

If postulate analysis was a genuine effort to introduce greater precision into mathematics, then it may appear to have been little more than a passing fad, of far less influence than other similar projects.^97^ But perhaps some subtle influence has acted on twentieth-century mathematics: the various group theory texts cited above may not give a prominent position to the postulational method, or cite any of the postulate analysts, but most of them do feature some elementary manipulation of postulates, or at least the derivation of simple consequences of them. We have already noted Carmichael’s removal of ‘certain redundancies’ to arrive at the one-sided definition of a group. Zassenhaus (1937, §1) similarly presented more than one set of axioms for a group (for example, our Definition 1 and also the definition by means of solvability in the group of the equations $ax=b$ and $ya=b$ for any *a*, *b*). Unlike the postulate analysts, he did not assert that one definition was any ‘better’ than another, he simply showed that the different definitions were equivalent, as a way of introducing his reader to elementary reasoning from defining postulates. The matter of independence is nowhere to be found nor is the vexed issue of ‘minimality’ and what it ought to mean. This is the extent of the postulational work that one typically finds in group theory books to this day: postulates are not studied for their own sake, but their manipulation serves as an introduction to method. This, I argue, is a legacy of the postulate analysts.

## A Huntington’s 1904 postulates

### A.1 Abelian groups

List of postulates I–II, *A*1–*A*6 defining an Abelian group, together with interleaved elementary theorems, taken from Huntington (1905a, §2):
Postulate I. There is an entity which belongs to the class.
Postulate II. If *a* is an element of the class, there is an element *b* such that $a\ne b$.
$$\ldots $$

*Theorem* 1. *There are at least two distinct elements in the class.*

Postulate *A*1. If *a* and *b* are elements of the class, then $a + b$ is an element of the class.
Postulate *A*2. If $a, b, a + b$, and $b + a$ are elements of the class, then

$$\begin{aligned} a+ b= b+ a. \end{aligned}$$

$$\ldots $$

Postulate *A*3. If $a, b, c, a+ b, b+ c, (a + b)+ c$, and $a+ (b+ c)$ are elements of the class, then

$$\begin{aligned} (a+ b)+ c=a+(b+c). \end{aligned}$$

$$\ldots $$

*Theorem* 6.^98^ *(a) The operation* $+$ *is always possible within the class, and it obeys (b) the commutative law and (c) the associative law.*

Postulate *A*4. If $a, x, y, a +x$, and $a + y$ are elements of the class, then from $a+x= a+y$ follows $x=y$.
$$\ldots $$

*Theorem* 7. *A change in either* *a* *or* *b* *alone produces a change in* $a + b$.
Postulate *A*5. If there is any element in the class, then there is an element 0 such that $0 + 0 = 0$.
$$\ldots $$

*Theorem* 8. *(a) There is a uniquely determined element* 0 *such that* $0+ 0 = 0$; *(b) for every element* *a*

$$\begin{aligned} a + 0=a\ \text {and}\ 0 + a = a; \end{aligned}$$

*and (c) if* $a + x = a$ *or* $x + a = a$, *then* $x= 0$.
$$\ldots $$

Postulate *A*6. If there is a uniquely determined element 0 such that $0+0=0$, then for every element *a* there is an element ${\bar{a}}$ such that $a+{\bar{a}}= 0$.
$$\ldots $$

*Theorem* 9. *(a) Every two elements*, *a* *and* *b*, *determine uniquely a third element* *x*, *denoted by* $b- a$, *such that*

$$\begin{aligned} a+(b-a)=b; \end{aligned}$$

*(b) in particular,*

$$\begin{aligned} a-a=0; \end{aligned}$$

*and (c) the element* $0-a$ *is usually abbreviated into* $- a$, *so that*

$$\begin{aligned} a+ (- a) = 0. \end{aligned}$$

### A.2 Arbitrary groups

List of postulates *G*1–*G*10 defining a group (an Abelian group if *G*11 is also included), together with interleaved elementary theorems, taken from Huntington (1905a, §5); as above, Postulates I and II are assumed, whence the above Theorem 1 also holds here.
Postulate *G*1. If *a* is an element of the class, then *aa* is an element of the class.
Postulate *G*2. If $a\ne b$, then *ab* is an element of the class. $\ldots$

*Theorem* II. *If* *a* *and* *b* *are elements of the class, then* *ab* *is an element of the class.*

Postulate *G*3. If there is any element in the class, there is an element *i* such that $ii =i$.
Postulate *G*4. If *x* and *y* are elements such that $xx= x$ and $yy= y$, then $x=y$. $\ldots$

*Theorem* III. *There is a uniquely determined element* *i* *such that* $ii=i$.
Postulate *G*5. If $ii=i$ and $aa\ne a$, then $ai = a$.
Postulate *G*6. If $ii=i$ and $aa\ne a$, then $ia = a$. $\ldots$

*Theorem* IV. *For every element* *a* *we have* $ai =ia =a$.
Postulate *G*7. If $ax=a$ and $aa\ne a$, then $xx=x$.
Postulate *G*8. If $xa = a$ and $aa\ne a$, then $xx = x$. $\ldots$

*Theorem* V. *If* $ax =a$, *or* $xa =a$, *then* $x=i$.
Postulate *G*9. If $aa\ne a, bb\ne b, cc\ne c$, and $ab\ne a, ab\ne b, bc\ne b, bc\ne c$; then

$$\begin{aligned} (ab)c=a(bc). \end{aligned}$$

*Theorem* VI. *For all elements* $(ab) c =a (bc)$. $\ldots$

Postulate *G*10. If $ii = i$ and $aa\ne a$, then there is either an element $a_r$ such that $aa_r =i$, or an element $a_l$, such that $a_la =i$.
$$\ldots $$

*Theorem* VII. Every element *a* determines uniquely an element ${\bar{a}}$ such that $a{\bar{a}}= {\bar{a}}a= i$.
*Theorem* VIII. A change in either *a* or *b* alone produces a change in *ab*.
$$\ldots $$

Postulate *G*11. If $aa\ne a, bb\ne b, ab\ne a, ab\ne b, ba\ne a$, and $ba\ne b$, then

$$\begin{aligned} ab= ba. \end{aligned}$$

$$\ldots $$

*Theorem* IX. For all elements $ab =ba$. $\ldots$
(NB. Postulate *G*10 in the above was explicitly acknowledged by Huntington as having been inspired by Moore’s six-postulate definition of a group in Moore (1902b); see Huntington 1905a, p. 35, footnote *.)

## B An approach to groups through triadic relations

In an address delivered to the Department of Mathematics of the International Congress of Arts and Science in St Louis, Missouri, in September 1904, Maxime Bôcher advocated the study of relations between the elements of a set, rather than that of operations on the set: Let us first notice that the equation $a\circ b = c$ denotes merely that the three objects *a*, *b*, *c* bear a certain relation to one another, say *R*(*a*, *b*, *c*). In other words the idea of an operation or law of combination between the objects we deal with, however convenient and useful it may be as a matter of notation, is essentially merely a way of expressing the fact that the objects combined bear a certain relation to the object resulting from their combination. Accordingly, in a purely abstract discussion like the present, where questions of practical convenience are not involved, we need not consider such rules of combination. (Bôcher 1904, p. 126) A footnote attached at the end of the above goes on: Even from the point of view of the technical mathematician it may sometimes be desirable to adopt the point of view of a relation rather than that of an operation. This is seen, for instance, in laying down a system of postulates for the theory of abstract groups (cf., for example, [Huntington 1901–1902b]), where the postulate:    If *a* and *b* belong to the class, $a\circ b$ belongs to the class, which in this form looks indecomposable, immediately breaks up, when stated in the relational form, into the following two:

1. If *a* and *b* belong to the class, there exists an element *c* of the class such that *R*(*a*, *b*, *c*).
2. If *a*, *b*, *c*, *d* belong to the class, and if *R*(*a*, *b*, *c*) and *R*(*a*, *b*, *d*), then $c = d$.

Huntington (1905b, p. 183) deemed this approach to be of ‘considerable logical interest’; given a class *K* and a relation *R* upon it, he set out the following postulates (Huntington 1905b, p. 192):
Postulate I. The class *K* is not an empty class.
Postulate II. Given *b* and *c* there is an *a* such that *R*(*abc*).
Postulate III. Given *a* and *c* there is a *b* such that *R*(*abc*).
Postulate IV. Given *a* and *b* there is a *c* such that *R*(*abc*).
Postulate V. If *R*(*abp*), *R*(*pcM*), *R*(*bcq*), and *R*(*aqN*), then $M= N$. From the above postulates, Huntington was able to deduce the following theorems:
*Theorem* 1. If *R*(*abc*) and $R ( a' bc)$, then $a =a'$.
*Theorem* 2. If *R*(*abc*) and $R(ab'c)$, then $b = b'$.
*Theorem* 3. If *R*(*abc*) and $R(abc')$, then $c = c'$. He concluded: If now we write $ab = c$ in place of *R*(*abc*), postulate V gives us the associative law: $(ab) c = (p) c = M; a (bc) =a (q) = N$; and all the conditions in Weber’s definition of a group are clearly satisfied. As in his other approach to groups, Huntington sought to prove the independence of the above system of postulates; he was able to do this for all the postulates except IV: ‘the question of the independence or deducibility of postulate IV is undecided’ (Huntington 1905b, p. 192). In light of this, he presented an alternative definition of a group by means of a triadic relation, ‘less symmetrical $\ldots$, but admitting complete proofs of independence’ (Huntington 1905b, p. 193):
Postulates I–III. The same as [above].
Postulate IV $'$. If *R*(*abc*) and $R ( abc')$, then $c = c'$.
Postulate V $'$. Either (1): If *R*(*abp*), *R*(*bcq*), and *R*(*pcM*), then *R*(*aqM*); or else (2): If *R*(*abp*), *R*(*bcq*), and *R*(*aqN*), then *R*(*pcN*). Huntington noted that the above Theorems 1–3 still hold for this new definition and that we also have the following:
*Theorem* $3'$. Given *a* and *b* there is a *c* such that *R*(*abc*). Huntington deduced thereby that his two definitions were equivalent and proved the independence of the postulates in the second. He also made the following comments, which signpost a direction for future investigation that he does not appear subsequently to have taken: It may be noticed that the postulates I–III, IV, and V$'$ are independent, but not sufficient to define a group, as witness the systems in which *R*(*abc*) is always true. The postulates I–III, IV$'$, and V are likewise independent, but their sufficiency is still an open question. (Huntington 1905b, p. 193) An investigation of the nature of the system (I, II, III, IV, V$'$) could have led to an analysis similar to that carried out for a different system by Clifford (as outlined in Appendix C), although this would likewise have gone beyond the realms of group theory.

## C Left and right groups

Our focus in the present paper has been on the study of group axioms, although, as noted in Sect. 5, other objects have received the postulational treatment. In most instances, the investigation of postulates has served as an exploration of the mathematical construction in question, as we have seen here for groups. However, on very rare occasions, ‘axiomatic tinkering’ has in fact resulted in new objects that have gone on to see further study. In this appendix, we describe one such instance that is related very closely to groups.

In Hollings (2014b, §6.4),^99^ I described how the first academic publication of the American algebraist A. H. Clifford (1933) had concerned a simple axiomatic question: the resolution of an apparent ambiguity in the group definition given by van der Waerden (1930, vol. 1, p. 15). Clifford began by writing down the following list of postulates:

(I): if $$a,b\in G$$, then $$ab\in G$$;
(II): for all $a,b,c\in G, a(bc)=(ab)c$;
(III): for each $a\in G$, there exists at least one left identity $e\in G$: $ea=a$;
(IV$$_L $$): for each $$a\in G$$ and each left identity *e* of *a*, there exists at least one left inverse *b* of *a*, with respect to *e*: $$ba=e$$;
(IV$$_R $$): for each $$a\in G$$ and each left identity *e* of *a*, there exists at least one right inverse *b* of *a*, with respect to *e*: $$ab=e$$;
(V$$_L $$): for each $$a\in G$$, there exists at least one left identity *e* of *a* and at least one left inverse *b*, with respect to *e*: $$ba=e$$;
(V$$_R $$): for each $$a\in G$$, there exists at least one left identity *e* of *a* and at least one right inverse *b*, with respect to *e*: $$ba=e$$.

The ambiguity in van der Waerden’s text lay in the fact that (Clifford claimed) his axiom relating to inverses could be interpreted either as (IV$$_L $$) or as (V$$_L $$). As Clifford noted: He evidently intended the former, judging from subsequent deductions; nevertheless I thought it would be of interest to see what the weaker postulate would lead to. (Clifford 1933, p. 866) Thanks to Dickson, Clifford knew that the system (I, II, III and IV$$_L $$) forms a group, for this is none other than our Definition 2: the ‘one-sided’ definition of a group. The description of the nature of the systems (I, II, III, X), where X is one of IV$$_R $$, V$$_L $$ or V$$_R $$ was his goal. Indeed, he showed immediately that these three systems are in fact one and the same (Clifford 1933, Theorem 1), a structure that Clifford dubbed a *multiple group*, and which he proved further to be left cancellative (Clifford 1933, Theorem 2). Moreover, every left identity has a group ‘around it’; these groups, which are mutually isomorphic, partition the multiple group. Since the left identities of a multiple group form a right zero semigroup (a semigroup in which $$ef=f$$, for any elements *e*, *f*), a multiple group is a union of groups that is indexed by a right zero semigroup, or, to put this another way, a direct product of a group and a right zero semigroup. Thus was born a very productive area of semigroup research: the investigation of those semigroups that are unions of groups; for further comments on this, see Hollings (2014b, §6.4). Multiple groups still appear in semigroup theory, but usually under the name *right group*, a name coined by A. K. Sushkevich (see Hollings 2014b, §6.3) when he had studied these objects (in terms of the structure of their ideals) in the finite case. In general, a right group is defined in a manner that is equivalent to that above: as a semigroup *S* that is both left cancellative and ‘right simple’, where the latter means that $$aS\cup \{a\}=S$$, for any $$a\in S$$. This last condition may of course be recast in a form that is reminiscent of postulates that we saw earlier in this paper: the equation $$ax=b$$ is solvable for any $$a,b\in S$$. A *left group* is similarly a semigroup that is right cancellative and left simple. Indeed, when one begins to look for them, one finds left and right groups scattered throughout the mathematical literature: Sushkevich and Clifford were just the first members of a long line of authors who (re)discovered their structure independently. In this appendix, we provide a (non-exhaustive) discussion of these various independent characterisations of left/right groups, considering the motivations of the different authors concerned.^100^

### C.1 General literature

As in Clifford’s case, the discovery of the structure of left or right groups has often been linked to questions about combinations of ‘lefts’ and ‘rights’ in the axioms. For instance, we find an example of a left group in D. E. Littlewood’s *The theory of group characters and matrix representations* of 1940; this appears simply as a demonstration of the fact that such a system is not a group; in a footnote appended to the definition of a group, he comments (concerning the identity element *I* and an arbitrary group element *a*): It is sufficient to assume ...  that $Ia=a$, and ... that $a^{-1}a=I$ ... The alternative pair of assumptions $aI=a, a^{-1}a=I$, however, is not sufficient. They hold for the set of 2-rowed matrices of the form $$\left[ \begin{matrix} a, &{} 0 \\ b, &{} 0 \end{matrix}\right] $$, where $$I=\left[ \begin{matrix} 1, &{} 0 \\ 0, &{} 0 \end{matrix}\right] $$, and these do not form a group, elements having, in general, no right-hand inverse. (Littlewood 1940, p. 32, footnote †)^101^
The first author to approach the characterisation of left groups after Clifford seems to have been the Slovak algebraist Štefan Schwarz in his 1943 paper ‘Theory of semigroups’ (‘Teória pologrúp’: Schwarz 1943). This is a paper that built up (largely independently of any other authors) a great deal of basic semigroup theory, particularly that concerning ideals of semigroups. In much of the paper, Schwarz confined his attention to semigroups in which every element has finite order, that is, every element has only a finite number of distinct powers (this is now termed a *periodic* semigroup: see Howie 1995, p. 12). Amongst other things, Schwarz extended a result of Sushkevich by showing that in such a semigroup, any minimal left/right ideal is a union of isomorphic groups. More specifically, he showed that every minimal left ideal ${\mathfrak {l}}$ may be written as a union ${\mathfrak {l}}=\bigcup _{\alpha \in A}{\mathfrak {G}}_\alpha$, for ${\mathfrak {G}}_\alpha =e_\alpha {\mathfrak {l}}$, where the $e_\alpha$ are the idempotents of ${\mathfrak {l}}$, indexed by some set *A* (Schwarz 1943, Theorems 19 and 20). For more on Schwarz’ algebraic work, see Hollings (2014b, §8.2).

From the year after Schwarz’s paper was published, we find another independent characterisation of right groups, this time in full generality, and due to H. B. Mann (1944) in a paper entitled ‘On certain systems which are almost groups’. Without any preamble (a feature common to many of the brief papers characterising left/right groups), Mann launched into the study of a set ${\mathfrak {S}}$ of elements together with a closed binary operation, subject to the conditions of associativity, existence of a left identity *E* for all elements and existence of right inverses with respect to *E*. To such a system, Mann gave the name *left right system*, or (*l*, *r*) *system* for short. He evidently knew that such a system need not be a group, and set out to determine its structure, which he approached via its idempotents. He noted, for example, that the idempotents of any (*l*, *r*) system themselves form an (*l*, *r*) system, which he called an *idempotent (l, r) system* (Mann 1944, Proposition 3). This is a right zero semigroup. The characterisation of an (*l*, *r*) system as the direct product of a group and an idempotent (*l*, *r*) system follows (Mann 1944, Theorem 2). Mann concluded with a nice example of an (*l*, *r*) system, quite different from previous examples. Let $S_1,\ldots ,S_n$ be statements, and let $A_i$ be the statement: “All the preceding statements are annulled but $S_i$ is true”. As Mann observed, the statements $A_i$ form an idempotent (*l*, *r*) system under conjunction of statements (Mann 1944, p. 881).^102^

The characterisation of right groups was also approached by K. Prachar in 1947 through the modification of group axioms (Prachar 1947). He began by listing the axioms given by Speiser (1923) and asked (i) how can they be modified in order that a group still results, and (ii) what other structures can result? Prachar gave some references to instances of these other structures, for example, Brandt’s groupoids (in the case where we no longer insist that the multiplication be defined everywhere: see Hollings 2009a, §4, or Hollings 2014b, §6.2), and quasigroups (where associativity is weakened: see Pflugfelder 2000). Prachar’s motivation for studying this problem seems to have come ultimately from a 1935 group theory lecture given by Philipp Furtwängler and attended by Prachar’s doctoral supervisor Edmund Hlawka. Furtwängler asked whether a group can be defined in terms of a left identity and right inverses. Hlawka later commented: I could solve this problem very easily by a counterexample, but the matter was not pursued because Furtwängler showed no particular interest in this solution.^103^
Hlawka eventually passed the problem on to Prachar, who gave a counterexample (a right zero semigroup of order *n*) to show that the answer to Furtwängler’s question is ‘no’; he also characterised any such system as a disjoint union of isomorphic groups.

The approach of Robert Ballieu (1950) was of a slightly different character and appears to follow in the traditions of the French semigroup school in the prominent use of equivalence relations (see Hollings 2014b, Chap. 7). Let *D* be a semigroup and define a relation *E* on *D* by the rule that $a\, E\, b$ if and only if $xa=xb$, for all $x\in D$. Then, *E* is a congruence on *D*. We may also consider the analogous relation $E_1$ on $D_1=D/E$, as well as $E_2$ on $D_2=D_1/E_1$, and so on. Ballieu gave examples to show that anything may happen with regard to the possible isomorphism of each term in the sequence with part or all of its predecessors. As a special case, he considered a semigroup *D* with at least one right identity, and such that every element of *D* has at least one left inverse with respect to at least one right identity. He proved that *D* / *E* is a group and that the semigroup ${\bar{e}}$ of all right identities of *D* is an equivalence class of *E*. He showed further that $D={\bar{e}}\times (D/E)$. A similar approach (via equivalence relations) was later adopted, independently, by Bengt Stolt (1953, 1956b) in his PhD thesis and a subsequent paper.^104^

In a short exploratory work on semigroups (Skolem 1951), the Norwegian mathematician Thoralf Skolem proved that any ‘associative semigroup with left division’ (a semigroup in which the equation $ax=b$ has precisely one solution for any elements *a*, *b*) is the direct product of a group and a ‘left unit system’ (a left zero semigroup) (Skolem 1951, Theorem 3). Skolem’s main interest here, however, was in semigroups with cancellation. After demonstrating that left division follows from left cancellation in the finite case (thereby giving a characterisation of any finite left cancellative semigroup as a direct product of a group and a ‘left unit system’), he next turned his attention to the infinite case with the comment: It remains, however, to study the infinite semi-groups with left cancellation rule. The treatment cannot be made complete here, because the number of possibilities is too great. (Skolem 1951, p. 45) The paper concludes with some very tentative first steps into the study of infinite left cancellative semigroups.

In ‘On a generalization of groups’, Hiroshi Hashimoto (1954) studied the following axioms for a group *G*:

(I): $$(ab)c=a(bc)$$;
(II): $$(a^\theta a)b=b$$;
(III$$'$$): $$a^\theta a=b^\theta b$$,

where $^\theta$ denotes an anti-automorphism of *G*, essentially inversion. Almost immediately, he replaced (III$'$) by the axiom:

(III) $$(ab)^\theta =b^\theta a^\theta $$.

Hashimoto termed any system satisfying (I), (II) and (III) a *G*-*system*. A group is then a *G*-system with $a=a^{\theta \theta }$. Hashimoto noted that $G^\theta =\{a^\theta :a\in G\}$ is a group and showed that *G* is the product of $G^\theta$ and the subset of idempotents of *G*.

The contribution of the Hungarian mathematician Jenő Szép is rather unusual amongst the papers surveyed in this appendix in that Szép provided motivation for his work (Szép 1956). Szép was interested principally in certain factorisations of groups,^105^ and he began his paper with the observation that any group *G* can be decomposed as $G=HC'$, where *H* is a subgroup of *G* and $C'$ is a subset of *G* with $H\cap C'=\{1\}$. It follows further that if *G* is a group and *H* a subgroup of *G*, then there exists a subset $C''$ of *G* such that $G{\setminus } H=HC''$ and $H\cap C''=\emptyset$. In the paper in question, Szép turned his attention to the derivation of a semigroup version of this decomposition. Let *F* be a semigroup and let *a* be any element of *F*. We put $[a]=\{a,a^2,\ldots \}$. Whenever it exists in *F*, we denote by $\{a\}$ the cyclic subgroup of *F* generated by *a*; we write the identity in $\{a\}$ as $e_a$. Let $\sqrt{a}$ denote an arbitrary solution to the equation $x^2=a$. We now define the following subsemigroup of *F* for each $a\in F$:

$$\begin{aligned} F_a= \left\{ \begin{array}{ll} \{a\}, &{}\quad \text {if this exists in } F \text { and all } \sqrt{a} \text { commute with } e_a; \\ {[}a{]}, &{}\quad \text {otherwise}. \end{array}\right. \end{aligned}$$

The main result of Szép’s paper states that, by analogy with the group case, if, for all $a\in F, F{\setminus } F_a$ admits a factorisation $F{\setminus } F_a=F_aC$, for some subset *C* with $F_a\cap C=\emptyset$, then *F* may be decomposed as $F=G_1\cup G_2\cup \ldots$, where the $G_i$ are pairwise disjoint subgroups of *F* whose identities $e_i$ satisfy the rule $e_ie_j=e_j$. Conversely, if a semigroup *F* admits the latter decomposition into disjoint subgroups, then each $F{\setminus } F_a$ admits the former decomposition.

L. N. Shevrin (1966) gave a characterisation of left groups in terms of the notion of a *left*/*right idealiser*: given a semigroup *S* with subsemigroup *T*, the *left*/*right idealiser* of *T* is the greatest subsemigroup of *S* containing *T* as a left/right ideal. He showed that a left group may be characterised as a semigroup in which every left idealiser of every cyclic subsemigroup is contained in some subgroup (Shevrin 1966, Theorem 3). E. G. Shutov (1966), much of whose semigroup-theoretic work concerned embeddings (see Hollings 2014b, §5.5), studied a related problem. In the course of studying left cancellative semigroups, Shutov proved, for example, that a semigroup is a right group if and only if it is regular (in the sense of Howie 1995, p. 50) and left cancellative. There is a small overlap between Shutov’s work and that of S. Lajos, whose publications list contains a significant number of papers on the structure of right groups (see, for example, Lajos 1964, 1966).

### C.2 Problems

Axiomatic studies related to left/right groups have also appeared in the problems pages of *The American Mathematical Monthly* and *The Mathematical Gazette*. In the former, for example, we find the following problem posed (in the non-associative case) by Goldman (1965): A groupoid is a nonempty set closed under a binary operation written multiplicatively. The direct product $G=G_1\times G_2$ of two groupoids consists of the Cartesian product of their sets together with component-wise multiplication. Suppose *G* admits such a factorization in which $G_1$ has the property that, if $x,y\in G_1$ then $xy= y$, while $G_2$ has a two-sided identity. Let *L*(*G*) consist of the left identities of *G*. Prove that $G_1\cong L(G)$ and that $G_2\cong Ge$ for some $e\in L(G)$. (Goldman 1965) This problem was solved in 1966 by a number of people (see Goldman and Thomas 1966). In a 1968 issue of *The American Mathematical Monthly*, we find the following, submitted by Peter Baxandall (1968): Let *S* be a semigroup with a right identity *e* (so $xe=x$ for all $x\in S$) such that given any $y\in S$ there exists ${\bar{y}}\in S$ with ${\bar{y}}y=e$. It is well known that *S* may not be a group. Is this still true if *S* has only one right identity? The problem had in fact been printed a year earlier (Baxandall 1967), but had appeared with a misprint: right inverses were specified instead of left! A negative solution due to Simeon Reich was published in 1969, along with a long list of names of other people (including Baxandall himself) who had also submitted solutions (see Baxandall and Reich 1969).^106^

Turning to *The Mathematical Gazette*, a related problem and solution were submitted by F. Gerrish in 1973; he asked whether inverses remain unique in a ‘group’ if we drop the condition of associativity and demonstrated that the answer is ‘no’. This was followed by a contribution from D. J. and E. R. Baylis (Baylis and Baylis 1973), who noted that ‘another question is naturally raised by this: is there anything “less drastic” than lack of associativity that will also give non-unique inverses?’ (Baylis and Baylis 1973, p. 208). They suggested the ‘less drastic’ method of permitting more than one (left or right) identity and gave the extreme example of a left zero semigroup, in which every element is a right identity. They went on: This observation led to a rough survey of about twenty books on group theory, with the following results. The majority defined both inverses and identities to be two-sided. The rest assumed the existence of only a left identity and left inverses, deduced the existence of their right partners, and proved their uniqueness and the equality of right and left partners. (Baylis and Baylis 1973, p. 209) They commented that D. E. Littlewood was the only author they could find who acknowledged the possibility of interest to us here. They proved that his example (see Appendix C.1) does indeed have the required properties, which then suggested to them the following result: if a semigroup *S* has a right identity *r* which is not a left identity, and all elements of *S* have a left inverse with respect to *r*, then there is an element with more than one right inverse. Their proof takes a mere five lines.^107^

Baylis and Baylis followed this up a couple of years later with a note with the intriguing title ‘A property of groups—via an impossible structure’ (Baylis and Baylis 1975). They noted here that it is perhaps beneficial to define a group by one-sided axioms and then to prove their two-sidedness and uniqueness, ‘since the proofs demonstrate at an early stage the power of the associative axiom’ (Baylis and Baylis 1975, p. 45). This note apparently stemmed from an attempt to remove the phrase ‘with respect to *r*’ from the result described in the preceding paragraph. However, Baylis and Baylis noted that ‘[t]he following rather surprising theorem explains why the attempt was doomed to failure’ (Baylis and Baylis 1975, pp. 45–46): there is no semigroup satisfying the conditions (A) there is a unique right identity *e*; (B) *e* is not a left identity; (C) each element has a left inverse with respect to *e*.

Baylis and Baylis here acknowledged the prior work of Mann and noted that an easy consequence of their theorem is that a ‘left right system’ is a group if and only if it contains a *unique* right identity and that every element has a left inverse relative to this identity.

## Footnotes

1 In the interests of varying language, I take ‘postulate’ and ‘axiom’ as synonyms, although the postulate analysts used ‘postulate’ quite consistently. For the most part, I deliberately overlook the fact that the two words have (or originally had) distinct meanings: a ‘postulate’ was an arbitrary supposition, whilst an ‘axiom’ was an assumption based upon an empirical source (see also the comments in Huntington 1937, p. 484). I would suggest, however that, following their experimentation with postulates, the postulate analysts appear to have gained some intuition for which postulates were sensible ones to take, and which were not (in this connection, see the Bell quotation in Sect. 6.1 beginning ‘What is wanted $\ldots$’): thus, we might argue that certain of the conditions that they used to define groups started as postulates and became axioms.

2 Easton was referring specifically to the definitions of Huntington and Moore that we will meet in Sect. 4.

3 I hope also that the present paper will complement another recent study of postulate analysis, namely Barnett (2016); whereas the focus of Barnett’s article is on an *individual* who worked in this area (E. V. Huntington), mine is on a particular *topic*.

4 We can of course make this definition considerably more formal through the use of predicate calculus, but we do not do so, since this is not the language that was used by any of the authors whom we will consider here.

5 In this situation, closure will usually fail because the result of applying the binary operation to two elements of the set under consideration may lie outside the set: the integers do not form a group under division, for example, because the result of dividing one integer by another may not be an integer. We note, however, that in this example, the result ‘1 / 2’, say, does nevertheless *exist*—the problem for our purposes is simply that it lies outside our predetermined set of objects of interest. This suggests therefore that there might be another way in which an operation may fail to be closed: through the non-existence of the required products, or, to put this a better way, through certain products being undefined (‘1 / 0’, perhaps?). Taking this point of view, we see that this is precisely the way in which the composition in a category, or in a related structure such as a groupoid, fails to be ‘closed’. This also brings up interesting questions about how to define a binary operation: considering (for simplicity) a category *C* in its ‘generalised monoid’ guise (on the two ways of viewing a category, see Hollings 2012, p. 417), we see that the operation in *C* is not simply a mapping $C\times C\rightarrow C$ because it will not, in general, be defined for all pairs in $C\times C$, merely for some subset $D\subseteq C\times C$. However, our algebraic instincts still lead us to view this operation as a ‘binary operation’ in some sense. We may perhaps resolve this difficulty by taking the binary operation not to be a *partial* mapping on $C\times C$ (that is, a mapping defined on a subset of $C\times C$), but rather a *fully defined* mapping on *D*. But here we find ourselves slipping into semantics, not to mention mathematical topics that lie outside our focus. On the development of category theory, see Corry (1996, 2nd ed., Chap. 8); on groupoids, see Brown (1987, 1999). For a discussion of the difficulties encountered prior to the eventual acceptance of partial operations, see Hollings (2014b, §10.2).

6 The reduction of the definition of an Abelian group to a single axiom not formed from the conjunction of simpler statements was achieved by Tarski (1938); a similar characterisation for an arbitrary group was obtained by Higman and Neumann (1952). These investigations were, however, considerably less elementary than those of Huntington *et al.*, so we do not consider them here.

7 In a later paper, Scanlan (2003) links the postulate analysts to the development of model theory, via the work of Alfred Tarski.

8 Indeed, if one adopts the point of view, noted after Definition 1, that a group is best regarded in the context of permutations, rather than as an abstract object, then Definition 1 has already taken us away from groups of permutations by explicitly featuring conditions other than closure.

9 An overview of some of the definitions presented in Sect. 2 may also be found in van der Waerden (1985, pp. 147–154).

10 For example, Franci (1992), which covers some of the same ground as our Sect. 4 and stands as a useful survey of the various definitions to be treated here.

11 I will quietly ignore the fact that the terms ‘permutation’ and ‘substitution’ (or, rather, their French equivalents) were not always employed with their modern meanings in the original texts. In his initial study of permutations, published in 1815, Cauchy used ‘permutation’ to denote a static arrangement of letters; the transition from one such arrangement to another was a ‘substitution’ (Cauchy 1815; see Stedall 2008, p. 354). This convention was later adopted by Galois (Stedall 2008, p. 362), but then dropped by Cauchy in his 1845 return to permutations, where both ‘permutation’ and ‘substitution’ were used for what had previously been dubbed ‘substitutions’ (Cauchy 1845a, pp. 595–596; see also Stedall 2008, p. 372).

12 Cauchy (1845a, p. 605); translation by Stedall (2008, p. 372). Note that Cauchy’s term ‘système de substitutions conjuguées’ has also been translated as ‘system of conjoined substitutions’ (Neumann 2011, p. 296), and ‘system of conjugate substitutions’ (Wussing 1969, English transl., p. 132), although the latter is probably best avoided, given the use of the word ‘conjugate’ in modern group theory. For further discussion of this translation, see Neumann (2008, pp. 175–176).

13 It is worth noting in passing that both Cauchy and Galois collected permutations into ‘systèmes’ and ‘groupes’, respectively, long before there was any formal notion of ‘set’.

14 To see a modern proof of this, suppose that $A=\{\alpha _1,\alpha _2,\ldots ,\alpha _n\}$ is a finite set of permutations of some set *X*, and suppose further that *A* is closed under composition, that is to say, *A* forms a semigroup. An elementary result about finite semigroups is that every element has an idempotent power (Howie 1995, Proposition 1.2.3). Since the only idempotent permutation is the identity permutation *I*, we conclude from the closure of *A* that $I\in A$. Furthermore, we have that for each $i, \alpha _i^{k_i}=I$, for some $k_i\in \mathbb {N}$, so each $\alpha _i$ has inverse $\alpha _i^{k_i-1}$.

15 This has subsequently been well documented by other authors: see, for example, Chakraborty and Chowdhury (2005), Katz (2009), Katz and Parshall (2014) and Neumann (1999), amongst many others.

16 On which, see, for example, Loe and Beagley (1997).

17 Further (circumstantial) evidence that Cayley still had ‘symbols of operation’ in mind is found in the fact that this prototype Cayley table is the mirror image (in the leading diagonal) of our modern equivalent: left-hand factors label the columns, and right-hand factors the rows—suggestive of a right-to-left composition of functions?

18 For example, the nonzero integers under addition obviously have the closure property of Cayley’s initial definition, but don’t satisfy the left/right multiplication property. Any semigroup *S* with the property that *S* is reproduced upon multiplication of the whole semigroup by an arbitrary element on the left/right is said to be *right*/*left simple*. These properties will appear again in Sect. 5.2.1 and are central also to the investigations outlined in Appendix C.

19 In the interests of balance, we note that a *defence* of Cayley may be found in Chakraborty and Chowdhury (2005).

20 ‘Es seien $\theta ',\theta '',\theta ''',\ldots$ Elemente in endlicher Anzahl und so beschaffen, dass sich aus je zweien derselben mittels eines bestimmten Verfahrens ein drittes ableiten lässt. Demnach soll, wenn das Resultat dieses Verfahrens durch ${\mathfrak {f}}$ angedeutet wird, für zwei beliebige Elemente $\theta '$ und $\theta ''$, welche auch mit einander identisch sein können, ein $\theta '''$ existieren, welches gleich: ${\mathfrak {f}}(\theta ',\theta '')$ ist. Überdies soll:

$$\begin{aligned} {\mathfrak {f}}(\theta ',\theta '')= & {} {\mathfrak {f}}(\theta '',\theta ')\\ {\mathfrak {f}}(\theta ',{\mathfrak {f}}(\theta '',\theta '''))= & {} {\mathfrak {f}}({\mathfrak {f}}(\theta ',\theta ''),\theta ''') \end{aligned}$$

und aber, sobald $\theta ''$ und $\theta '''$ von einander verschieden sind, auch:

$$\begin{aligned} {\mathfrak {f}}(\theta ',\theta '')\text { nicht identisch mit }{\mathfrak {f}}(\theta ',\theta ''') \end{aligned}$$

sein.’ (Kronecker 1870, pp. 882–883; note that as a concession to typesetting in , the long s used in places by Kronecker has been replaced by its short equivalent.)

21 ‘*Definition.* Ein System *G* von *h* Elementen irgend welcher Art, $\Uptheta _1,\Uptheta _2,\ldots ,\Uptheta _h$ heisst eine *Gruppe vom Grade h*, wenn es den folgenden Bedingungen genügt:

I.: Durch irgend eine Vorschrift, welche als Composition oder Multiplication bezeichnet wird, leitet man aus zwei Elementen des Systems ein neues Element desselben Systems her. In Zeichen $$\begin{aligned} \Uptheta _r\Uptheta _s=\Uptheta _t. \end{aligned}$$
II.: Es ist stets $$\begin{aligned} (\Uptheta _r\Uptheta _s)\Uptheta _t=\Uptheta _r(\Uptheta _s\Uptheta _t)=\Uptheta _r\Uptheta _s\Uptheta _t. \end{aligned}$$
III.: Aus $\Uptheta \Uptheta _r=\Uptheta \Uptheta _s$ und aus $\Uptheta _r\Uptheta =\Uptheta _s\Uptheta$ folgt $\Uptheta _r=\Uptheta _s$.’ (Weber 1882, p. 302)

22 Suppose that *S* is a finite cancellative semigroup. It follows immediately from cancellation that $sS=S=Ss$, for any $s\in S$. From this, we conclude that, for a fixed *s*, there exists $e\in S$ such that $se=s$. But *e* is in fact a right identity for any element of *S*, since any $t\in S$ may be written in the form $t=us$. It follows further that $e^2=e$. Consider the product *es*, for any $s\in S$. We may write *s* as *eu*, for some $u\in S$. Then, $es=e^2u=eu=s$. Thus, *e* is a two-sided identity for *S*. As to inverses, it follows from $sS=S$ that there exists $s'\in S$ such that $ss'=e$. Furthermore, $ss's=es=s=se$, and so it follows from cancellation that $s's=e$.

23 ‘Ein System *P* von Dingen (Elementen) irgend welcher Art wird zur Gruppe, wenn folgende Voraussetzungen erfüllt sind:

1.: Es ist eine Vorschrift gegeben, nach der aus einem ersten und einem zweiten Elemente des Systems ein ganz bestimmtes drittes Element desselben Systems abgeleitet wird. $\ldots$
2.: das associative Gesetz vorausgesetzt, $\ldots$
3.: Es wird vorausgesetzt, dass, wenn $ab=ab'$ oder $ab=a'b$ ist, nothwendig im ersten Falle $b=b'$, im zweiten $a=a'$ sein muss.’ (Weber 1895, vol. II, pp. 3–4)

24 ‘Für endliche Gruppen ergiebt sich aus 1., 2., 3. die Folgerung:

4.: Wenn von den drei Elementen *a*, *b*, *c* aus *P* zwei beliebig gegeben sind, so kann man das dritte immer und nur auf eine Weise so bestimmen, dass $$\begin{aligned} ab=c \end{aligned}$$ ist.’ (Weber 1895, vol. II, p. 4)

25 ‘Für unendliche Gruppen kann nicht mehr so geschlossen werden. Für unendliche Gruppen wird also noch die Eigenschaft 4. als Forderung in die Begriffsbestimmung mit aufgenommen.’ (Weber 1895, vol. II, p. 4)

26 Within the development of the abstract group concept, Neumann (1999) gives an indication of who read whose work.

27 See footnote 11 on the distinction between permutations and substitutions.

28 For suppose that $ga=gb$, for $a,b\in G$ with $a\ne b$—but then $|gG|<|G|$.

29 On the empirical aspects of choosing postulates, see the comments in footnote 1.

30 To pick just one example, see, for instance, the detailed references given in the footnotes on p. 182 of Huntington (1905b).

31 I will not describe the origins of postulate analysis in great detail here; instead, see Corry (2000, §2) (or Corry 1996, 2nd ed., §3.5), together with Bell (1938), Scanlan (1991) and Schlimm (2011). See also the wider discussion in Zitarelli (2001). On Hilbert’s *Grundlagen der Geometrie*, see Hallett and Majer (2004).

32 On Pierpont (1866–1938), see Ore (1939).

33 ‘Die in diesem Theil entwickelten Sätze gelten für alle Gruppen, die aus einer *endlichen* Anzahl von Operationen bestehen. Die Art der Operationen ist dabei gleichgültig. Es wird nur die Gruppeneigenschaft vorausgesetzt, welche in die folgenden Bestimmungen zusammengefasst werden kann:

1): Je zwei Operationen sollen in bestimmter Aufeinanderfolge zusammengesetzt (multiplicirt) eine eindeutig bestimmte Operation ergeben, welche gleichfalls derselben Gesammtheit angehört.
2): Für die Zusammensetzung der Operationen soll das associative Gesetz gelten, während das commutative nicht erfüllt zu sein braucht.
3): Aus jeder der beiden die Operationen *A*, *B*, *C* enthaltenden symbolischen Gleichungen $$\begin{aligned} AB=AC,\quad BA=CA \end{aligned}$$ soll geschlossen werden können, dass $$\begin{aligned} B=C \end{aligned}$$ ist.

Eine Folge dieser Bestimmungen im Zusammenhang mit der Endlichkeit der Operationenzahl ist es, dass eine sogenannte *identische* Operation *J* vorhanden ist, und zwar eine einzige, welche alle anderen bei der Multiplication unverändert lässt, und dass zu jeder Operation *A* eine eindeutig bestimmte umgekehrte Operation $A^{-1}$ sich findet, so dass

$$\begin{aligned} AA^{-1}=A^{-1}A=J \end{aligned}$$

ist.’ (Hölder 1889, pp. 28–29)

34 We note that although Hölder gave no direct source for his notion of group, a footnote within the passage quoted above directs the reader to ‘see also’ Dyck (1882) ‘[r]egarding the group definition’ (‘Hinsichtlich der Gruppendefinition vergl. auch $\ldots$’: Hölder 1889, p. 29).

35 The review, dated December 1897, appeared in the February 1898 issue of the *Bulletin of the American Mathematical Society*; the two Galois theory articles are dated, respectively, February and August 1900, although it is not clear exactly when those particular issues of the *Annals of Mathematics* appeared in print. As a further piece of information regarding dating, we record the following footnote from the first page of Pierpont (1899–1900): ‘The following pages consist of a reproduction, with slight alterations, of parts of a course of lectures delivered in September 1896 at the *Buffalo Colloquium* held under the auspices of the American Mathematical Society’.

36 Take, for example, the concluding paragraph of the review (Pierpont 1898, p. 234): ‘We will close now our review regretting its inadequacy to give but a faint idea of the merits of this great work. A classic from the day of its publication, it is destined to a long and useful career, a monument of honor to its genial author’.

37 Indeed, many years later, Øystein Ore asserted that Pierpont’s lectures on Galois Theory were ‘obviously inspired by Kronecker’s and Weber’s expositions’ (Ore 1939, p. 481).

38 This paper was presented to a meeting of the AMS in New York on 22 February 1902 and published later that same year. Indeed, it was one of three papers that Huntington presented at this AMS meeting; the other two also concerned systems of postulates: ‘A complete set of postulates for the theory of absolute continuous magnitude’ and ‘Complete sets of postulates for the theories of positive integral and positive rational numbers’ (see Kasner 1902, pp. 273–274).

39 Huntington (1901–1902a, p. 298). It is perhaps of interest to reproduce Huntington’s proof of this lemma (where we retain his numbering of equations: ($1^\circ$), ($2^\circ$), etc.). Using postulate 1, we take *e* such that $a\circ e = a$ ($1^\circ$), and $b'$ such that $b\circ b'=e$ ($2^\circ$). By postulate 2, we may take *c* such that $c\circ b'=a$ ($3^\circ$). By postulate 1 again, we take $\beta$ such that $a\circ \beta =c$ ($4^\circ$), and $\beta '$ such that $\beta \circ \beta '=e$ ($5^\circ$). It follows from ($3^\circ$) and ($4^\circ$) that $(a\circ \beta )\circ b'=a$ and from ($1^\circ$) and ($5^\circ$) that $a\circ (\beta \circ \beta ')=a$. By postulate 3, $(a\circ \beta )\circ \beta '=(a\circ \beta )\circ b'$. Therefore, by left cancellation, $\beta '=b'$. Using this, ($5^\circ$) becomes $\beta \circ b'=e$, so that, by ($2^\circ$), $\beta \circ b'=b\circ b'$. Therefore, by right cancellation, $\beta =b$. It follows from ($4^\circ$) that $a\circ b=c$, as required.

40 To return to the musings of footnote 5, we note that there is never any doubt that the result of applying Huntington’s rule of combination to two elements from the assemblage *exists* (in some sense—the assemblage is implicitly assumed to be sitting inside some universal set)—the question, at least initially, is of where that result lies.

41 For example, the positive integers under the operation $a\circ b=a$ satisfy 2 and 3, but not 1.

42 A point not remarked upon by Huntington is the fact that this fourth postulate is of a rather different character from the preceding three. In modern language, it either takes Huntington’s system out of the class of objects that are first-order definable, or else it embeds it in another theory (namely, set theory) where finiteness is definable.

43 We note a further minor difference: the ‘or’ in his earlier version of associativity became ‘and’ here. Whereas before, $a, b, c, a\circ b$ and $b\circ c$, together with either of $(a\circ b)\circ c$ or $a\circ (b\circ c)$, were assumed to belong to the set in question, now the assumption covered all of these elements (implicitly for *a* and *b*). Huntington probably noticed that, in fact, the equality in the earlier version ensured (trivially) that $(a\circ b)\circ c$ and $a\circ (b\circ c)$ must both belong to the set, and so the phrasing could be simplified. We will see in Sect. 4.3 that Moore seized upon the ‘or’ of Huntington’s earlier associativity condition as a means of breaking the postulate down into simpler parts.

44 See the comments in Moore (1902b, p. 485, footnote §); see also footnote 43 of the present paper.

45 Another very minor peculiarity of the definition lies in the postulates ($5_\alpha$) and ($5_\beta$): Moore distinguished between different *finite* orders, but not between different *infinite* orders. However, although the distinction between different finite orders became important later on when Moore came to enumerate the number of steps needed to verify different sets of postulates, this was not something that he could extend to the infinite case, so there was no need for him to distinguish between different infinite orders.

46 Indeed, see the comment in Sect. 3 on closure as ‘the group property’.

47 Around the same time, Moore included further musings on the preferred nature of postulates in his presidential address to the AMS (Moore 1903, pp. 403–405).

48 Curiously, Moore had postulates labelled (3) and (3$''$), etc., but no (3$'$), etc.—perhaps the latter had appeared in an earlier draft of the paper but was deleted without the numbering being changed?

49 Namely, $N^3$ ways of selecting the three elements *a*, *b*, *c*, followed by the calculation of four products: *ab*, *bc*, (*ab*)*c* and *a*(*bc*).

50 Huntington (1905a, p. 24) also gave brief consideration to the ideas of ‘categorical’ vs. ‘disjunctive’ sets of postulates, concepts that he borrowed from Veblen (1904, §2): a set of postulates is *categorical* if it determines an object that is unique up to an appropriate notion of isomorphism, and *disjunctive* otherwise; any set of group postulates is necessarily disjunctive, given that there may be at least two non-isomorphic groups for a particular order.

51 With regard to the six-postulate definition of an Abelian group that Huntington gave here (see Appendix A.1), Franci (1992, p. 273) notes that Huntington probably gave such a long definition, abandoning his previous drive towards ‘minimal’ definitions, because
$\ldots$ he realized that for some purposes more axioms give a more immediate information [*sic*] about the structure, as in this case, where his next step is to use the axioms of abelian groups to define the structure of the real number field.

52 Cf. the comment in Sect. 3 on closure as ‘the group property’.

53 Cf. the way in which closure is specified in Cayley’s ‘definition’ of a group in Sect. 2.2.

54 A further piece of evidence that practical considerations were not necessarily at the forefront of Huntington’s mind when he developed his systems of axioms is the fact that his Postulate I in the paper under consideration (see Appendix A.1) rules out the possibility of the trivial Abelian group $\{0\}$. Since this is rather an important Abelian group, it appears that, for Huntington, this list of postulates was indeed merely a definition and that he had not thought through the consequences of using it as a practical starting point for doing group theory.

55 See, for example, Huntington (1916, 1924b, 1935), and Huntington and Kline (1917).

56 We note in passing that Huntington’s brief investigation of ‘triadic-relation’ postulates for groups led him to a system of axioms that, although independent, did not in fact define a group (see Appendix B). The study of postulates for a different ‘non-group’ by A. H. Clifford (outlined in Appendix C) led to some early results in the theory of semigroups, but Huntington did not attempt any further analysis of his system, suggesting that his focus was very much upon the investigation of *groups* and that he had little or no interest in studying other objects that might arise through ‘axiomatic tinkering’.

57 See, for example, the references in Sect. 5.1.

58 In fact, Dickson (1905a, p. 199) began with another over-elaborate form of closure, specifically the three postulates:

($1_1$): For every two equal or distinct elements *a* and *b* of the set, there is a determination of $a\circ b$.
($1_2$): For *a* and *b* in the set, there is at most one determination of $a\circ b$.
($1_3$): If, for *a* and *b* in the set, there is at least one determination of $a\circ b$, one determination is an element of the set.

However, he noted immediately that these may be combined into the ‘triple statement’:

1. For every two equal or distinct elements *a* and *b* of the set $a\circ b$ is uniquely determined as an element of the set.

59 Nowadays, the term ‘semigroup’ is of course used for a more general concept: a set with a (closed) associative binary operation. On the place of this work of de Séguier and Dickson in the development of the theory of semigroups, see Hollings (2009a, §3) or Hollings (2014b, §1.2).

60 Some Ukrainian postulational work that will not be dealt with here but that is still worth mentioning is that of S. O. Shatunovskii: see Hollings (2014a).

61 See, for example, Bell (1927a) or Stone (1935), as well as the treatments of this topic in Mehrtens (1979, §1.2) and Barnett (2016).

62 See Ward (1930), Garver (1934, 1935, 1936), Boggs and Rainich (1937), Rabinow (1937a, b), and, a little later, Sánchez-Díaz (1953) and Furstenberg (1955).

63 See Vandiver (1934a, b, 1940a, b, 1952, 1953) and Vandiver and Weaver (1956, 1957, 1958).

64 An earlier contribution was made by Adolf (Abraham) Fraenkel in 1914, but we will not go into this here: see instead Corry (2000). On German contributions in this area, see also Barnett (2016, p. 13).

65 For brief biographical comments on Klein-Barmen, see Hollings (2014b, p. 348).

66 Indeed, Klein-Barmen is almost certainly the source of the use of this word in German to mean an abstract lattice. On Klein-Barmen’s place within the development of lattice theory, see Schlimm (2011); see also Mehrtens (1979, §3.2.2).

67 This treatment of Baer and Levi’s paper is adapted from that in Hollings (2014b, pp. 65–67), where it is considered within the context of the development of semigroup theory.

68 This definition of left/right simplicity is equivalent to that given in footnote 18.

69 See, for example, the comments in Wussing (1969, §III.4.4).

70 Schmidt (1916, 2nd ed., p. 9): ‘

1.: .
2.: $$\begin{aligned} A(BC)=(AB)C. \end{aligned}$$
3.: $$\begin{aligned} AI=A. \end{aligned}$$
4.: $$\begin{aligned} AX=I.\text {'} \end{aligned}$$

71 A comment in the preface to the second edition of Schmidt’s book suggests one possible influence: ‘My book grew out of the work of the seminar of Professor D. A. Grave, my teacher $\ldots$’ (Schmidt 1916, 2nd ed., p. 5: ‘’). It is entirely possible that the work of the postulate analysts was discussed in Grave’s seminar (which was held in Kiev), but more research is needed to determine whether this was the case and to investigate the influence of Grave’s seminar more generally (for a few very brief comments, see Hollings 2015, p. 196). In his own textbook on groups (Grave 1908), Grave confined himself largely to the finite case and to groups of transformations of various kinds. His abstract definition of a finite group (pp. 58–59) includes axioms for closure, associativity and (two-sided) cancellation; for the infinite case, he added the condition that in the equation $AB=C$, each of the three elements *A*, *B*, *C* is determined by the other two. We note that Schmidt’s distinctive term ‘symbolic multiplication’ (‘’) was employed by Grave also.

72 See, for example, Maltsev (1971), or the brief comments in Hollings (2015, §§1–2).

73 For a discussion of Sushkevich’s book within the context of the development of semigroup theory, see Hollings (2014b, §12.1.1).

74 Sushkevich (1937, p. 3): “”

75 See footnote 50.

76 See, for example, Hollings (2009b).

77 On the content of the dissertation more generally, see Hollings (2014b, §3.2).

78 Some of the remarks of E. T. Bell that appear in Sect. 6.1 were aimed at a general readership.

79 Barnett (2016, p. 11) notes that although the main protagonists in the story of postulate analysis were indeed American, we might nevertheless view postulate analysis as a subdiscipline of mathematical logic, whose European representatives influenced, and were cited by, the postulate analysts. She argues further for a subtle influence from postulate analysis on mathematical logic that served to cement the international reputation of the American mathematical community (Barnett 2016, p. 2).

80 But to be fair to Bell, we must record his comment that ‘the modern abstract algebra of the past two decades is still too close to us for an impartial appraisal’ (Bell 1938, p. 5).

81 We note that Birkhoff also wrote favourably of the postulational method elsewhere: see Birkhoff (1976).

82 Regarding Definition 1, Bell later commented: ‘The postulates contain redundancies but are more easily seen in [this] inelegant form’ (Bell 1952, p. 169).

83 Drawn from the Whitehead Library of the Oxford Mathematical Institute, the library of The Queen’s College, Oxford, and the author’s own bookshelves. The books sampled include textbooks, monographs, lecture notes and popular books, mostly focused on groups specifically, rather than algebra generally. They range in date from 1896 to 2012, and, although the majority are in English, others are in German, French, Portuguese and Russian.

84 I count those with and without closure together as a way of simplifying this analysis and ensuring that its focus is on the presence or absence of the identity and inverse axioms. Of the 72 books sampled, 33 (46%) mentioned closure explicitly. Unsurprisingly, associativity is present in all 72 definitions.

85 For instance, James (1963, p. 11) defines a group to be a nonempty set with an associative binary operation such that all left and right translations are bijective and then immediately deduces the existence of identity and inverses. Schenkman (1965, p. 2), on the other hand, defines a group to be a semigroup with a unary operation $^{-1}$ such that $aa^{-1}b = b = baa^{-1}$; he immediately proves that such a system contains an identity and that the unary operation is, of course, none other than the usual inverse operation. Stewart (1994, p. 43), on the other hand, gives Definition 1 and notes its redundancies, but remarks that ‘[i]n practice it is easier to stick to the axioms we have given’.

86 With a curious bias towards the right-hand version (7:2).

87 Indeed, this might even colour the learner’s view of different branches of mathematics in interesting ways. As a piece of anecdotal evidence from the author’s own experience, the view around 15 years ago amongst some mathematics students at the University of York was that group theory was a very axiomatic topic, but that analysis was not, owing to the ways in which these subjects had been introduced, and the features that had been emphasised: the axioms for groups, and $\varepsilon$s and $\delta$s for analysis. In contrast, current mathematics students at the University of Oxford begin their introductory analysis course with a detailed treatment of the axiom system for the real numbers and only learn about groups several months later. Thus, when asked to make certain deductions from the axioms for a vector space, one student remarked: ‘This is like an analysis proof’.

88 In the sense of footnote 50.

89 See, for example, the polemics Hammersley (1968) and Kline (1973); for a later account, see Walmsley (2003).

90 See, for example, the survey of criticisms of Bourbaki in Mashaal (2002, Chap. 9).

91 Outside the Bourbaki tradition, the work of Paul Dubreil may provide an interesting point of comparison with that of the postulate analysts; his textbook Dubreil (1946) was one of those in the above-mentioned sample that features a one-sided definition of a group, and contains some brief postulational considerations.

92 Despite the fact that van der Waerden’s definition of a group went on to spark the postulational investigations outlined in Appendix C.

93 Carmichael did this in preparation for a short discussion of the postulates for a linear associative algebra (Carmichael 1937, p. 397), a concept that would perhaps have been less familiar to his readers than that of a group.

94 A further example is provided by Miller *et al.* (1916, pp. 105, 160–161).

95 She comments, for example, that Huntington is most widely remembered today for his contribution to the study of mathematical methods of apportionment; the method of appointing representatives to the US Congress used today is due to him. (Barnett 2016, p. 4)

96 Wilhelm Magnus’ preface to the 1958 Dover reprint of Dickson’s book likewise makes no comment on Dickson’s omission.

97 Take, for example, the introduction of Weierstrassian rigour into analysis, to which Bell compared postulate analysis in some of his writings on the subject: see Hollings (2016, pp. 70–71).
